# Rqc1 and other yeast proteins containing highly positively charged sequences are not targets of the RQC complex

**DOI:** 10.1016/j.jbc.2021.100586

**Published:** 2021-03-24

**Authors:** Géssica C. Barros, Rodrigo D. Requião, Rodolfo L. Carneiro, Claudio A. Masuda, Mariana H. Moreira, Silvana Rossetto, Tatiana Domitrovic, Fernando L. Palhano

**Affiliations:** 1 Programa de Biologia Estrutural, Instituto de Bioquímica Médica Leopoldo de Meis, Universidade Federal do Rio de Janeiro, Rio de Janeiro, RJ, Brazil; 2 Programa de Biologia Molecular e Biotecnologia, Instituto de Bioquímica Médica Leopoldo de Meis, Universidade Federal do Rio de Janeiro, Rio de Janeiro, RJ, Brazil; 3 Departamento de Ciência da Computação, Universidade Federal do Rio de Janeiro, Rio de Janeiro, RJ, Brazil; 4 Departamento de Virologia, Instituto de Microbiologia Paulo de Góes, Universidade Federal do Rio de Janeiro, Rio de Janeiro, RJ, Brazil

**Keywords:** translation, ribosome, protein synthesis, translation control, ubiquitin ligase, ribosome quality control, ribosome profiling, poly (A) tracts, polybasic sequences, stalled polypeptides, CAT, C-terminal alanine-threonine, CHX, cycloheximide, CSC, codon stabilization coefficient, ICP, inhibitory codon pair, RP, ribosome profiling, RQC, ribosome quality control, TE, translation efficiency

## Abstract

Previous work has suggested that highly positively charged protein segments coded by rare codons or poly (A) stretches induce ribosome stalling and translational arrest through electrostatic interactions with the negatively charged ribosome exit tunnel, leading to inefficient elongation. This arrest leads to the activation of the Ribosome Quality Control (RQC) pathway and results in low expression of these reporter proteins. However, the only endogenous yeast proteins known to activate the RQC are Rqc1, a protein essential for RQC function, and Sdd1, a protein with unknown function, both of which contain polybasic sequences. To explore the generality of this phenomenon, we investigated whether the RQC complex controls the expression of other proteins with polybasic sequences. We showed by ribosome profiling data analysis and western blot that proteins containing polybasic sequences similar to, or even more positively charged than those of Rqc1 and Sdd1, were not targeted by the RQC complex. We also observed that the previously reported Ltn1-dependent regulation of Rqc1 is posttranslational, independent of the RQC activity. Taken together, our results suggest that RQC should not be regarded as a general regulatory pathway for the expression of highly positively charged proteins in yeast.

The first study to propose that the translation of positive amino acids could lead to translational stalling was published in 2008 by Lu and Deutsch (1). It was already known that the ribosomal exit tunnel, a narrow passage through which the nascent peptide passes as soon as it is synthesized, is negatively charged (2) and that the translation of the poly (A) tail from constructs lacking a stop codon, which leads to the incorporation of lysine residues (the codon AAA translates into lysine), causes translational repression (3, 4, 5). This knowledge led to the hypothesis that a high concentration of positively charged residues inside the exit tunnel could slow translation rates due to the presence of electrostatic interactions. Using a cell-free approach, Lu and Deutsch, 2008, demonstrated that the presence of lysine or arginine repeats in a protein sequence is sufficient to decrease translation rates. They observed that the presence of as few as four nonconsecutive arginines in the construct was already enough to diminish the translational speed, while an equal concentration of negatively charged or uncharged residues did not affect translation rates. This phenomenon was also observed with lysines and was independent of codon choice suggesting that the retardation effect was explicitly caused by the charge itself, not by the amino acid structure or tRNA availability (1). The ejection of nascent proteins out of the ribosome exit tunnel is also modulated by electrostatic interactions (6).

These early experiments prompted the use of polybasic sequences as stalling regions in many subsequent papers. Most of these publications focused on the characterization of the ribosome quality control (RQC), a cellular pathway responsible for the cotranslational degradation of nascent peptides (7, 8). Polybasic sequences were added to, or removed from, a reporter protein and the amount of translated protein was detected by western blot (7, 9, 10). Using this approach, several authors have confirmed that the presence of polyarginine (R), polylysine (K), or mixtures of both amino acids can reduce protein production (11, 12, 13) and that this effect was linked to the action of the RQC machinery (7, 14, 15, 16, 17).

The RQC complex removes stalled nascent polypeptides from the ribosome and activates stress signals in *Saccharomyces cerevisiae* (7, 17). A defective RQC complex causes proteotoxic stress in yeast cells (10) and neurodegeneration in mice (18). The components of the yeast RQC complex are Rqc1, Rqc2, and Ltn1 proteins, all of which have a relatively low number of proteins per cell when compared with the number of ribosomes per cell, thus indicating that the system could easily reach saturation (7, 14, 19, 20, 21). The RQC complex function is modulated by Asc1 and Hel2. These proteins detect ribosome stalling and promote early termination, leading to ribosomal subunit dissociation and transcript degradation (7). After ribosome dissociation, the RQC complex can bind to the 60S subunit and induce the degradation of the nascent polypeptide through the E3 ubiquitin ligase activity of Ltn1 (7, 9). Rqc1 and Ltn1 also recruit Cdc48 and its cofactors, Npl4 and Ufd1, which uses the energy from ATP hydrolysis to extract the stalled ubiquitylated polypeptide from the 60S subunit leaving the 60S subunit free to engage in another round of translation (8). Rqc2 is involved in stress signaling through the formation of the C-terminal alanine-threonine (CAT) tail. After attaching to the 60S subunit, Rqc2 can incorporate alanine and threonine residues to the C-terminal of the stalled peptide, creating the CAT tail (10, 22, 23, 24, 25). This incorporation is random and independent of mRNA, and it is important to promote protein aggregation and/or to expose lysine residues that may be trapped inside the ribosome exit tunnel to the ubiquitin ligase activity of Ltn1 (24).

It has been proposed that the trigger for RQC activity is ribosome collision, with the formation of disomes, trisomes, and even tetrasomes (15, 26, 27, 28, 29). However, recent studies on the global analysis of disome formation suggested that collisions are relatively common, and most of these events are resolved without translation termination (30). What determines whether a collision event will become a target of RQC or not is still an open question, but the disome structure, the time required to resume translation, and exposure of an empty A site seem to play an important role in this decision (31). Different studies have shown that the existence of stalling features in endogenous sequences seems to be accompanied by other unique characteristics, such as low initiation efficiency (32, 33) and/or the positioning of the stall sequence at the beginning of the transcript (34). Very recently, it was showed a *cis*-acting negative feedback loop mediated by translation inhibitors upon dissome formation (35, 36). These adaptations are thought to result in fewer ribosomes per transcript, which would avoid ribosome collisions at the stalling-prone segments, thus preventing RQC activation.

In addition to the action as a salvage pathway for ribosomes stuck in unproductive translation events, it was suggested that the RQC pathway could regulate the endogenous expression of transcripts containing stalling features. This idea was put forward by an observation that Rqc1 levels increase with the deletion of *ASC1*, *HEL2*, *LTN1*, or *RQC2*, suggesting that Rqc1 protein expression could be controlled by RQC activity, providing negative feedback for the system (7). Rqc1 contains a well-conserved K/R polybasic sequence in its N-terminal that, when substituted by alanine residues, led to increased protein expression. Based on these data, it was suggested that the RQC complex regulates Rqc1 protein levels through its polybasic sequence during Rqc1 translation (7). More recently, the protein Sdd1, which has a polybasic sequence in its C-terminal, has been reported as another target of the RQC complex. Similar to Rqc1, the protein expression of Sdd1 was increased with the deletion of proteins of the RQC complex or with mutations that substitute the positively charged residues from its polybasic sequence to alanine residues (26). However, polybasic sequences usually are RQC activators if coded by rare codons or poly-A-stretches, as recently revised in Requião *et al.*, 2020 (37). Indeed, the polybasic sequence of *SDD1* contains a CGA-CGA inhibitory codon pair (ICP) and a short poly (A) tract (26), which may explain its inhibitory property, but the polybasic sequence of Rqc1, regardless of the codon composition, is thought to be sufficient for translation termination and RQC recruitment (7). In summary, the influence of polybasic sequences, either alone or combined with poly (A) sequences or ICP, on RQC recruitment is not yet fully established.

In order to answer if other endogenous proteins with polybasic sequences would be subjected to RQC regulation, we first performed a bioinformatics screening to identify the yeast proteins containing polybasic sequences (sequences with the highest concentrations of K/R of the yeast proteome). Then, we analyzed previously published ribosome profiling experiments (26, 38, 39, 40, 41) to access the impact of polybasic sequences on the translation of these proteins. Ribosome profiling is based on the deep sequencing of ribosome-protected mRNA fragments (42). During translation, each ribosome encloses a portion of mRNA, protecting it against RNase digestion. This enclosure allows protected fragments to be sequenced, generating a map, at nucleotide resolution, of ribosomal occupancy on transcribed mRNAs. This map can report the occurrence of ribosome pausing or stalling, revealed by the accumulation of deep sequencing reads at specific positions of the transcript. We observed that polybasic sequences led to slower ribosome movement during translation and an increase in disome formation. However, we could not detect signatures of severe ribosome stalling, such as the presence of short (20–22 nt) mRNA fragments, which indicates empty A site in ribosomes (43). These data suggested that polybasic sequences of endogenous proteins do not necessarily lead to RQC complex activation, as previously described (37). To confirm that these sequences cannot hamper the translation of endogenous proteins, we selected genes containing extremely positively charged segments, and without altering their original promoter or their 5’ UTR, we tested their protein expression in yeast strains deleted for RQC components. In this experimental setting, we could not observe an increase in their steady-state protein levels, which is usually observed with reporters containing stalling sequences. These results suggest that RQC activity does not downregulate translation of endogenous proteins with polybasic sequences in general. Our data indicate that polybasic sequences of endogenous proteins are sufficient to slow ribosome movement, but are not sufficient to induce the recruitment of the RQC complex.

In the case of Rqc1, we could detect its upregulation in the *ltn1*Δ background, but its levels were unperturbed when the complex was disabled at upstream steps (*asc1*Δ, *hel2*Δ) or at the CATylation step (*rqc2*Δ). An important observation was that the deletion of *LTN1* increased the expression of the full-length Rqc1, suggesting that this regulation could not be coupled to a putative stalling event at a polybasic sequence located at the N-terminal. The half-life of Rqc1 protein was much shorter in wt cells when compared with *lnt1*Δ cells. These results are inconsistent with the current model of Rqc1 being regulated by the RQC complex. Based on these data, we conclude that the RQC machinery is mostly dedicated to recycling ribosomes involved in aberrant mRNA translation and plays a small role in controlling endogenous levels of polybasic proteins under normal growth conditions. Moreover, we propose that Rqc1 regulation by Ltn1 is a posttranslational event, independent of the RQC activity.

## Results

### Identification of polybasic sequences in the proteome of *S. cerevisiae*

Our first step was to find endogenous yeast proteins containing sequences with different arrangements of polybasic sequences. We selected four distinct but possibly overlapping architectures, covering most of polybasic sequence variations analyzed in previous studies: (a) sequences of eight or more lysines/arginines residues (K/R) in a window of ten amino acids, (b) six or more consecutive K/R, (c) adenine repeats longer than ten nucleotide residues, and (d) net charge ≥+12 in a window of 30 residues. The architectures a) and b) were selected because they are often present in RQC activation reporters and lead to the accumulation of reads in ribosome profiling data (7, 10, 22, 23, 24, 25, 27, 29, 44, 45, 46, 47, 48, 49, 50, 51, 52). The architecture c) was selected because poly (A) mRNA composition can lead to ribosome sliding, worsening the translation efficiency of these sequences (53, 54). Architecture d) was selected because proteins containing a stretch of 30 residues (approximately the polypeptide length enclosed by the ribosome exit tunnel) with net charge ≥ +12 are extremely rare in most proteomes (55, 56). We also compared these architectures with a group of 1800 genes containing ICPs that are known to impair translation with substantial downregulation in protein output (*e.g.*, CGA-CGA and CGA-CCG) (57). It is important to note that the ICP group, used herein as an endogenous positive control for ribosome stalling, has some potential pitfalls. For example, the sequence CGA-CCG-A, captured in our list of ICP as CGA-CCG (Table S1), likely induces frameshifting (58). Even though the pair CGA-CCG is present in just 1% of the genes in our list, we cannot rule out that other ICP arrangements might cause frameshifting and lead to poor translation scores by mechanisms unrelated to ribosome stalling.

**Figure 1:**
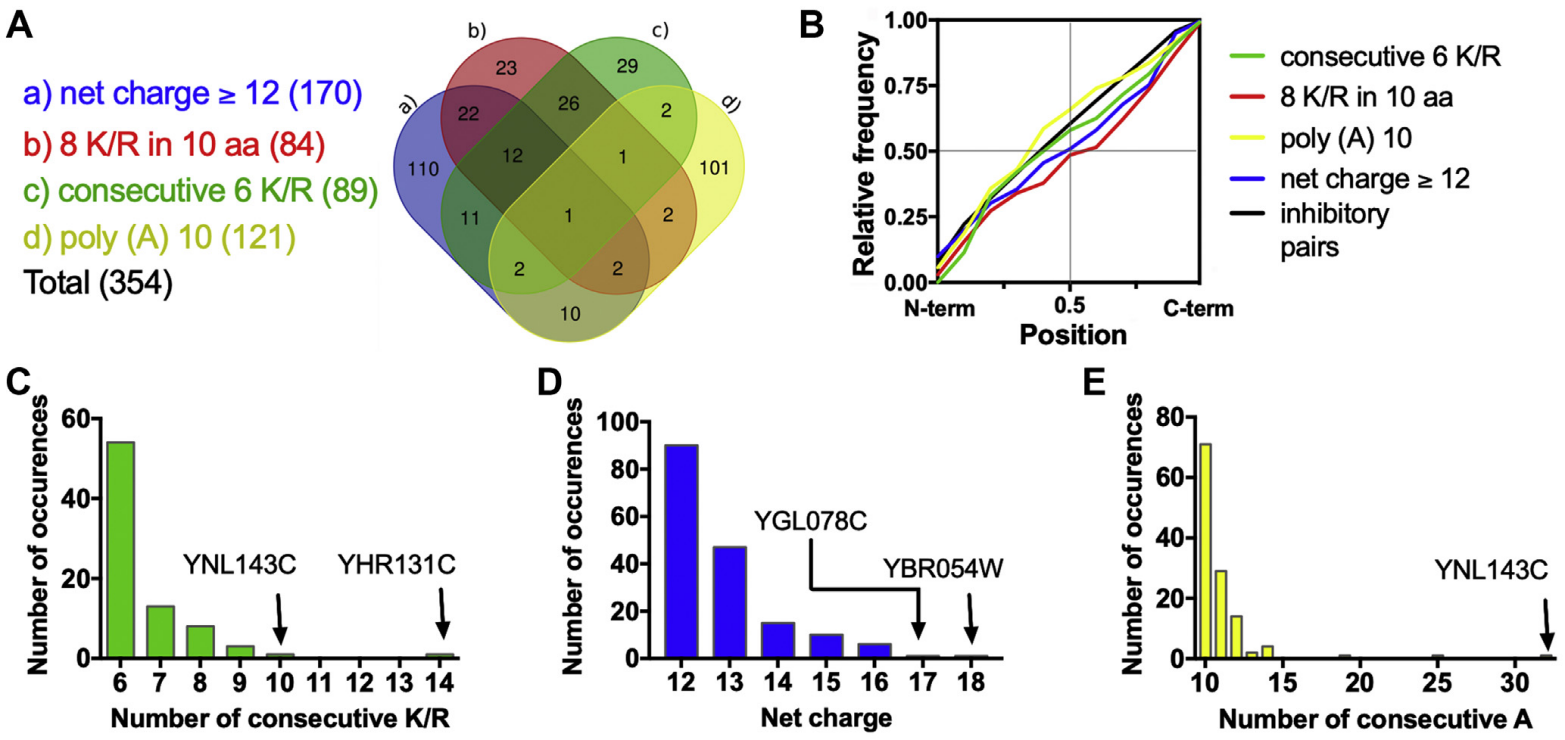
**Bioinformatics screening to identify the polybasic sequences in the yeast proteome.***A*, Venn diagram showing the genes identified as potential targets of the RQC complex. The list of identified genes is presented in Table S2. *B*, cumulative frequency distribution of the location of the polybasic sequences of each group. For each gene, the desired attribute was located, and its position was determined in relation to the length of the gene. The frequency of distribution of the genes is depicted in the following categories: (*C*) consecutive 6 K/R, (*D*) net charge equal to or higher than +12, and (*E*) ten or more consecutive adenines. The genes with the highest scores of each category were highlighted.

We found that 354 proteins (approximately 6% of the yeast proteome) contained at least one of the characteristics described above (Fig. 1*A*). The features most commonly found were high net charges and poly (A) sequences, with 170 and 121 sequences, respectively. Moreover, the Venn diagram (Fig. 1*A*) shows that just only 91 proteins have more than one feature at the same time, and only one protein, the uncharacterized YNL143C, has all the characteristics. Localization analysis revealed that no bias was found for the different categories analyzed (Fig. 1*B*). We selected YNL143C and other proteins with prominent polybasic sequences (Fig. 1, *C*–*E*) for further experimental analysis in the next sections.

### Proteins with polybasic sequences show divergent patterns of translation efficiency parameters

If the presence of polybasic sequences represents an important obstacle to translation, we expect to find signatures of poor translatability in proteins that contain them. For example, endogenous genes containing stall sequences have a slower or less efficient translation initiation; this is thought to be an adaptation to avoid ribosome collision during elongation (33). Therefore, we asked whether the polybasic and the ICP data sets would be significantly different from the rest of the yeast genes in previously published experimental measurements that reflect translation activity. We chose six parameters that indicate how well a transcript is translated: translation efficiency (TE), codon stabilization coefficient (CSC), time for translation initiation, Kozak score, Kozak score 50 nt, and 5’ UTR footprint density. The TE is a metric derived from ribosome profiling data, calculated from the ratio of ribosome-protected footprint read counts to the total mRNA read counts for each gene (59). The CSC is a metric derived from mRNA half-life that positively correlates with TE and protein abundance (60). The time for translation initiation is calculated from ribosome profiling density, relative mRNA concentration, and time of translation for individual codons (61). The Kozak score is determined by reporter expression quantification from a library containing synthetic 5 nt sequences cloned upstream the reporter’s start codon, while the Kozak score 50 nt is determined by reporter expression quantification from a library containing 5’ 50 nt UTR sequences from yeast genes cloned upstream the reporter’s start codon (62). Finally, the 5’ UTR footprint density is determined by ribosome profiling reads detected at 40S ribosomal subunits (63). To make this analysis, we used publicly available data sets generated by previous studies (59, 60, 61, 62, 63). The Table S3 shows how these metrics correlate with each other for the entire genome data set. TE had a positive correlation with CSC and both Kozak scores and negative correlation with time for translation initiation and 40S 5’ UTR densities. Moreover, even though we are using different ribosome profiling experiments to obtain the TE and time for initiation values, we still observed the expected positive correlation between these parameters (Table S3).

**Figure 2:**
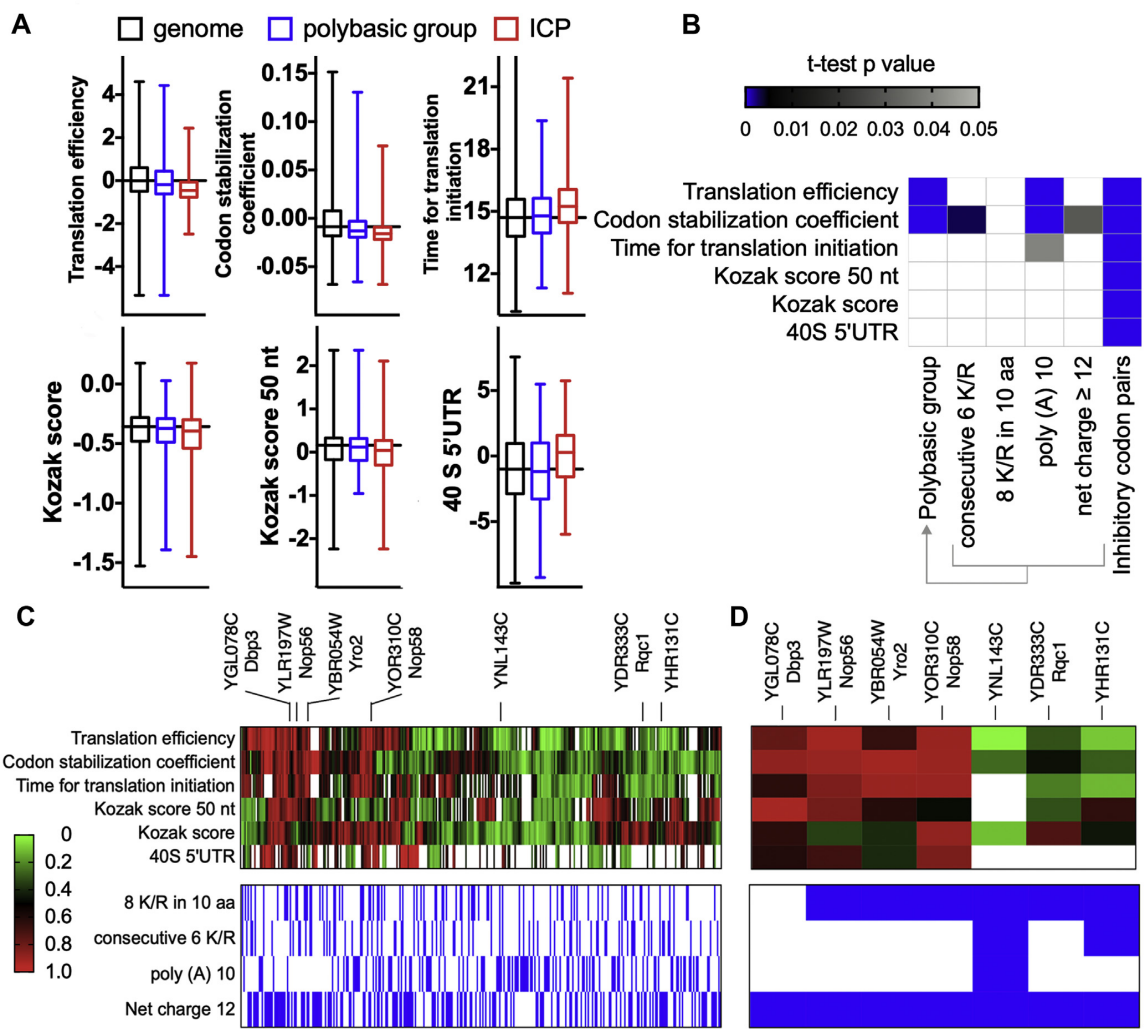
**Translatability scores of genes with polybasic sequences.** The different groups of genes with polybasic sequences were compared regarding parameters that, to some extent, reflect the efficiency of translation. *A*, box plot comparing different parameters from three groups of genes, namely genome, polybasic group, and inhibitory codon pairs (ICP). *B*, the Kolmogorov–Smirnov test *p* values are plotted for each comparison. The *white rectangle* means that a nonsignificant difference was found (*p* values > 0.05). As a control, genes with one of the 17 inhibitory codon pairs (ICP) were used. *C*, the values of the translation initiation parameters were normalized (where 0 and 1 represent the lowest and highest initiation rates of the full genome, respectively), and then the 354 identified genes were clustered by the Euclidean distance. The *lower panel* indicates the polybasic architecture present in each gene (marked in *blue*). *D*, translation initiation parameters cluster of the genes with the most prominent polybasic sequences (Fig. 1, *C*–*E*) and YDR333C/Rqc1 (included for comparison). The raw data, sample size, and *p* values are presented in Table S4.

In Figure 2*A*, we compare the distribution obtained with the entire genome with the distribution of values obtained with the ICP and polybasic gene groups. Consistent with the pattern expected for hard-to-translate sequences, the ICP group showed higher initiation times, higher 40S 5’ UTR densities, and lower TE, CSC, and Kozak scores. All these differences were supported by a *t*-test analysis comparing the individual data sets with the rest of the yeast genes (*p*-values are shown in Fig. 2*B*). For the polybasic group, the only differences with a significant *p*-value were lower TE and CSC values than the genome group. Separating the polybasic sequences into subgroups did not reveal a particularly deleterious architecture of polybasic sequence arrangement (Fig. 2*B*). The cumulative distributions for each of the measurements described above are shown in Fig. S1.

To evaluate the characteristics of each gene from the polybasic group, we created a unified rank for all the measurements above. Using the values obtained with the entire yeast data set, each score system was organized in ten bins; next, the maximum and minimum bin values were used to normalize the scale. We created a heat map (Fig. 2*C*, upper panel) where 1 refers to the maximum translation activity (high TE, high CSC) or optimal initiation (short translation initiation time, high Kozak's efficiency, and low 5’UTR footprint density of the 40S ribosomal small subunits). A cluster analysis of the 354 genes with polybasic sequences revealed that the group is rather heterogeneous and displays all spectra of characteristics, with some sequences having excellent translatability indicators with poor initiation scores and vice versa (Fig. 2*C*). The lower panel indicates the polybasic architecture present in each gene. Notably, the sequences with the highest positively charged sequences or longest poly (A) tracts also showed diverse features (Fig. 2*C*, lower panel). Therefore, we could not find a clear signature of poor translatability for genes containing polybasic sequences using the analyzed parameters (Fig. 2, *C* and *D*).

### Ribosome profiling of endogenous proteins with polybasic sequences

**Figure 3:**
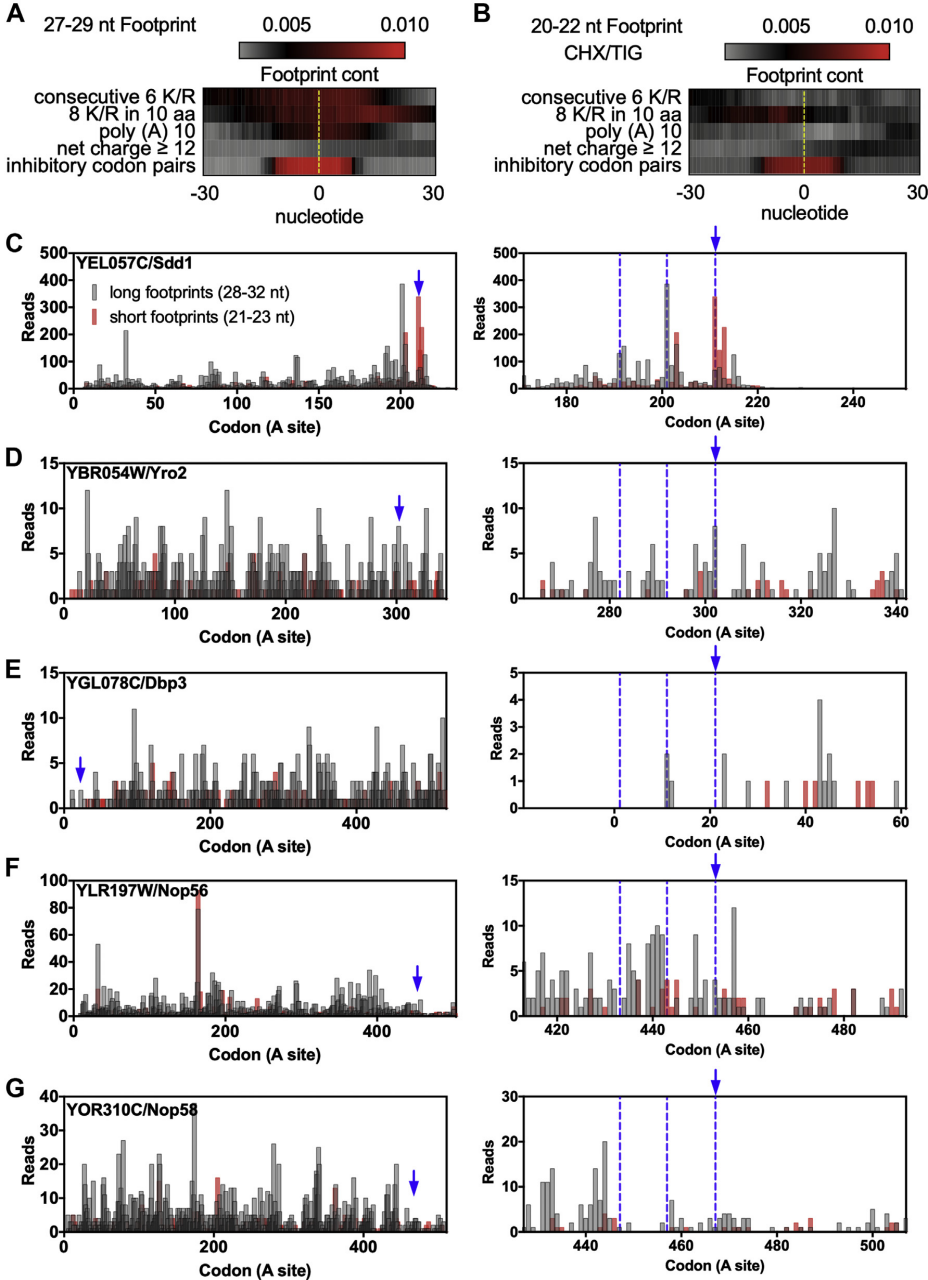
**Ribosome profiling data of polybasic sequences.***A*, 27 to 29 and (*B*) 20 to 22 nucleotide footprint analyses of genes containing polybasic sequences. As control, genes with one of the 17 inhibitory codon pairs (ICP) were used. The *dotted yellow line* represents the start of the polybasic sequence or of the ICP. *C*, 28 to 32 and 21 to 23 nucleotide footprint analysis of Sdd1 shows an accumulation of short reads at its polybasic site, indicating ribosome stalling, and accumulation of long reads upstream of the stalling site with a periodicity of roughly ten codons, indicating ribosome collisions, which are highlighted on the *right panel*. *Blue arrow* indicates the start of the polybasic sequences, and the *blue dotted line* indicates the hypothetical position of the collided ribosomes. *D*–*G*, 28 to 32 and 21 to 23 nucleotide footprint analysis of the proteins with the highest polybasic sequences (YHR131C and YNL143C were omitted because of their low RP coverage). For panels *A* and *B*, the full read was used while for panels *C*–*G*, just the ribosomal A site read was used. The reads were plotted at an approximate position of the ribosomal A site.

To test whether the selected polybasic sequences have any effect on translation, we again took advantage of previously published ribosome footprint profiling data (26, 38, 39, 40, 41), which were used on all subsequent ribosome profiling analyses. (please see Experimental procedures section Ribosome profiling data for detailed information). Analyses of the ribosome profiling (RP) data of the polybasic and ICP sequences indicated an enrichment of 27 to 29 nucleotide long footprints around the stalling sequence (Fig. 3*A*). We also plotted the number of reads at an approximate position of the ribosomal A site and observed an accumulation of reads downstream the first codon of the polybasic sequence (Fig. S2), which suggests a reduction in translational speed.

Additionally, we looked directly at the presence of ribosome collisions through the analyses of disome profiling. The disome profiling allows sequencing of mRNA fragments protected by two stacked ribosomes and shows stalling patterns undetected by traditional RP (30, 40, 42). Disome footprints could be detected as a sharp peak immediately after the ICP sequences (Fig. S3, *A*–*C*). The analysis of polybasic sequences also showed an increase in the disome footprints. However, the peak was broader than the one observed with ICPs and occurred 9 to 10 codons after the polybasic-coding nucleotides (Fig. S3, *D*–*F*). This observation supports the hypothesis that the arrest is caused by electrostatic interactions of the nascent positively charged polypeptide with the ribosome exit tunnel. Since disomes were detected in most transcripts, it is unlikely that the formation of these disomes alone can trigger the RQC activation (30, 41).

Recently, it was shown that slow decoding of the second codon of the ICPs results in an empty ribosomal A site, which leads to shorter 20 to 22 nucleotide long footprints in RP experiments and represents slow decoding rates. These shorter footprints correspond to ribosomes in a classical state (or post-state), which are waiting for the arrival of the next tRNA, different from ribosome in a rotated state (or pre-state), which results in longer 27 to 29 footprints (39, 43). Structural and biochemical evidences demonstrated that the electrostatic repulsion caused by the presence of a polybasic sequence inside the exit tunnel can decrease the binding efficiency of an incoming tRNA carrying an additional positively charged residue, which could also lead to empty A site ribosomes (26, 43, 64). To evaluate if the polybasic sequences of endogenous proteins can cause the accumulation of ribosomes in the classical state (open A sites), we looked for short footprints in RP data of these proteins and found no evidence. The ICP was the only group that showed an accumulation of short reads (Fig. 3*B*). The same result was observed with another RP data set obtained with a different combination of translation inhibitors (cycloheximide (CHX) and anisomycin) (Fig. S4). These results suggest that polybasic sequences can reduce elongation rates (Fig. 3*A*), but they are not enough to cause severe ribosome stalling leading to the accumulation of open A site ribosomes (Fig. 3*B*).

Another indicative of severe ribosome stalling is the occurrence of ribosome collisions upstream an empty A site ribosome (27, 29). If the endogenous polybasic sequences are strong stalling sequences, we should observe not only an increase in short nucleotide footprints corresponding to an empty A site, but also an increase in long nucleotide footprints upstream of the stalling site with a periodicity of roughly ten codons, which corresponds to the collided ribosomes (26). Among the 354 genes with one or more polybasic sequences identified in our bioinformatics screening, we selected the four top genes (Fig. 1, *C*–*E*) for subsequent analyses: YBR054W/*YRO2* (net charge = +17), YGL078C/*DBP3* (net charge = +16), YHR131C (14 consecutive arginines), and YNL143C (32 consecutive adenines coding for ten lysines) (Figs. 2 and 3). To expand our analyses, we also included genes with the two longest polybasic sequences, stretches with six or more K/R in ten amino acids of the yeast proteome: YLR197W/*NOP56* and YOR310C/*NOP58* (42 and 44 residues long polybasic sequences, respectively). As a positive control, we included the gene *SDD*1, which has been recently described as an endogenous target of the RQC complex and shows the ribosome collision footprint pattern described above (26). Our analyses revealed that, apart from *SDD*1, none of the selected genes had the periodic footprint pattern that would indicate ribosome collisions (indicated by dashed lines) (Fig. 3, *C*–*G*). A downside of this analysis was the low ribosome profiling coverage of the polybasic genes, and this is the reason why YHR131C and YNL143C were not included in Figure 3, *C*–*G*. In an attempt to enhance the analysis sensitivity, we carefully examined all the biological replicates looking for consistent read patterns that could suggest the presence of queued ribosomes. We also checked the ribosome profiling of a *hel2*Δ strain, where the stalled ribosomes are more stable and produce stronger signals corresponding to the periodic pattern of collided ribosomes than in the wt strain (Figs. S5–S9). *SDD1* presented a reproducible pattern across all the replicates and a stronger queued ribosomes signal in the *hel2*Δ strain (Fig. S5). However, no consistent periodicity or queued ribosomes signal increase in the *hel2*Δ strain could be observed for the polybasic genes analyzed (Figs. S6–S9). The ribosome collision footprint pattern was also carefully investigated for the remaining 348 proteins with polybasic sequences but, due to the low ribosome profiling coverage, no conclusion could be reached (data not shown).

In conclusion, we observed that the ICP group of genes produced more features associated to ribosome stalling than the polybasic group (Fig. 3 and Fig. S3). Therefore, polybasic sequences may represent a superable obstacle to translation under normal growth conditions, and their presence is not enough to induce RQC activation.

### The six yeast proteins with most prominent polybasic sequences are not targets of the RQC complex

**Figure 4:**
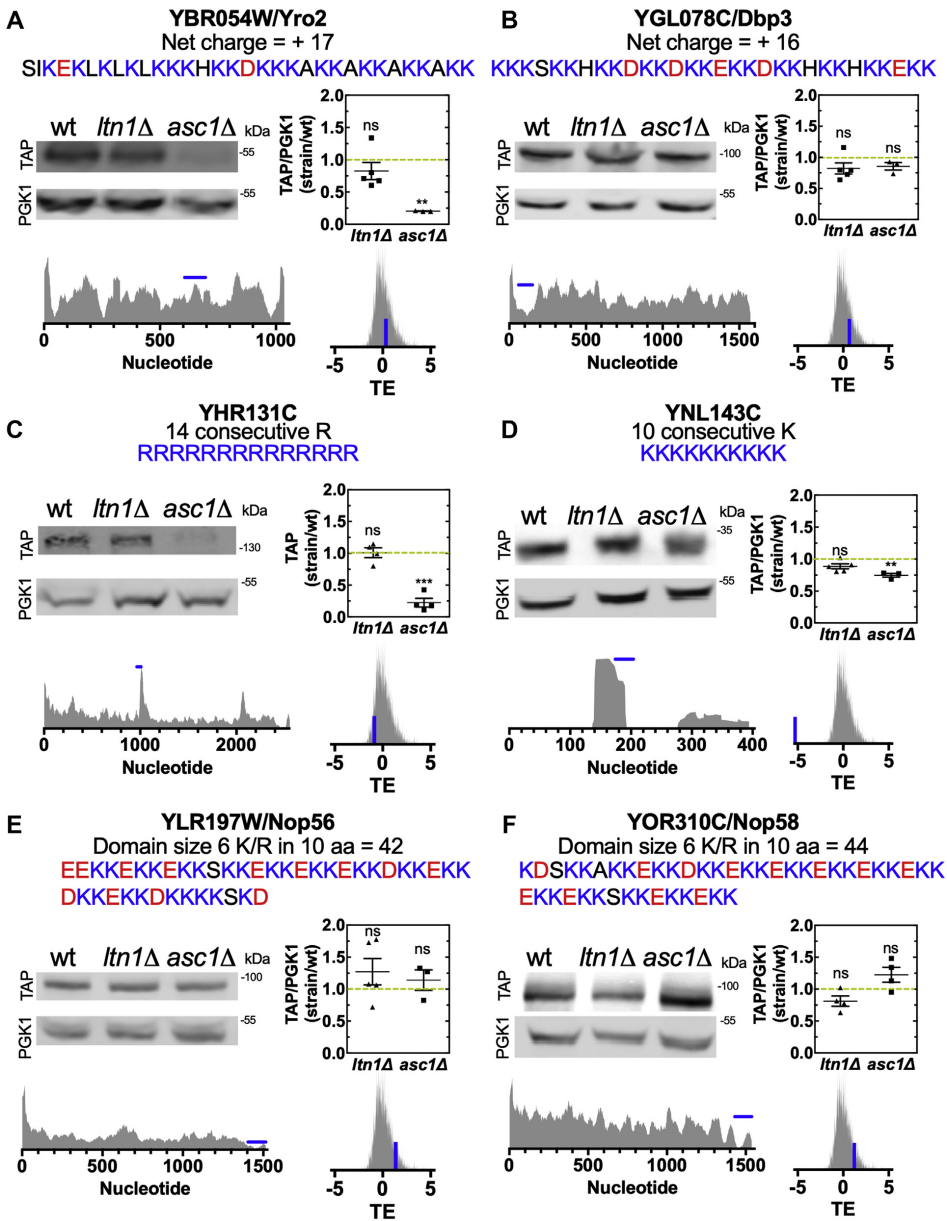
**Proteins with the greatest polybasic sequences are not targets of the RQC complex in yeast.***A*–*F*, proteins with the greatest polybasic sequences of yeast were measured by western blot in wild type (wt), *lnt1*Δ, and *asc1*Δ strains. For each protein, the primary sequence of their polybasic sequence, their ribosome profiling data, and translation efficiency (TE) are presented. The *blue bar* in the ribosome profiling data indicates the position of the polybasic sequence, while the *blue bar* in the TE data shows the TE of the specific gene inside the TE distribution of all genes. The band intensity was quantified and normalized in relation to the housekeeping protein phosphoglycerate kinase (Pgk1). One-way ANOVA with Bonferroni's multiple comparison test was used for statistical analyses.

To confirm that polybasic sequences are not sufficient to cause ribosome stalling and translation termination, we examined whether the deletion of RQC components had any effect on the final protein output of our selected proteins, which would imply a regulatory role for the RQC complex on their expression. TAP-tagged versions of the six genes selected from the previous section were obtained from a yeast library containing a chromosomally integrated TAP-TAG at the 3’ region of each gene, thus preserving the natural promoter region and the 5’ UTR of the transcripts (65). We chose to delete *LTN1*, the E3 ubiquitin ligase of the RQC, and *ASC1*, involved in the modulation of RQC activity because they represent different steps in the RQC pathway and because they have been consistently used in the investigation of RQC mechanism of action (7, 21, 26, 52, 66, 67, 68). Each TAP-TAG strain was used to generate the *ltn1*Δ or *asc1*Δ variants, which were confirmed by PCR and western blot (Fig. S10). In Figure 4, we show the protein expression of each gene as probed by anti-TAP western blot in the three different backgrounds and the relative quantification of at least three independent experiments. For each gene, we also show their ribosome profiling (complete sequence, with the polybasic sequence marked in blue) and their TE (depicted as a blue bar relative to total distribution of TE values of the yeast genes). We observed that deletion of *LTN1* caused no change in the protein levels of the selected polybasic proteins. Since the TAP-TAG is downstream the putative stalling sequence, the lack of Ltn1 should not stabilize the full-length construct. Although the results from the *ltn1*Δ strains may have a limited contribution for the understanding of the role of RQC in the regulation of the analyzed genes, it showed that these proteins are not regulated by Ltn1 in an RQC independent pathway, as we observed for Rqc1 (see next section).

The results with *asc1*Δ revealed no changes in YGL078C/Dbp3, YNL197W/Nop56, and YOR310C/Nop58 protein levels and a downregulation in YBR054W/Yro2, YHR131C, and YNL143C (Fig. 4). This pattern could not be explained by features such as ribosome density on the polybasic site, TE, or initiation time. Asc1 has been linked to the assembly of the translation preinitiation complex, and its deletion impairs translation of a subset of genes, particularly small ORFs and genes linked to mitochondria function (69, 70). These roles of Asc1 unrelated to RQC could be responsible for the decreased levels since YBR054W/Yro2 protein is localized to the mitochondria and YNL143C protein is encoded by a small ORF.

Nevertheless, all tested proteins differed from what was observed with reporters containing stalling sequences, which had their expression levels upregulated in *asc1*Δ strains (7, 21, 52, 66, 67, 68). Therefore, we conclude that, even though Asc1 positively regulates the expression of some of the proteins, the complete RQC pathway is not actively contributing to the regulation of the endogenous levels of any of the proteins tested.

### Ltn1 regulates Rqc1, but the mechanism is independent of the RQC complex

As described above, the proteins Rqc1 and Sdd1 have been previously reported as targets of the RQC complex. The regulation of Sdd1 showed the canonical pattern expected for a protein regulated by the RQC activity: deletion of *LTN1* increased the levels of the truncated N-terminal of Sdd1, while deletions of *ASC*1 or *HEL2* increased the levels of its full-length protein (26). On the other hand, the experimental data available on Rqc1 regulation showed increased levels of the full-length construct in an *ltn1*Δ background, when the expected result would be the stabilization of the N-terminal fragment up to the stalling polybasic region (7). This result is not consistent with a model of cotranslational regulation of Rqc1 by the RQC.

**Figure 5:**
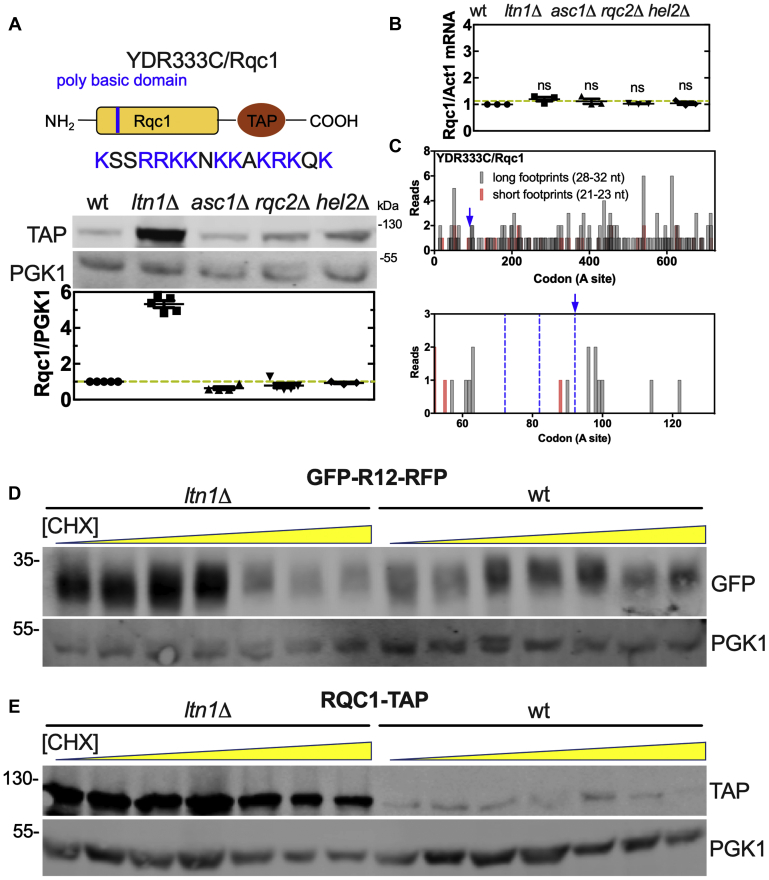
**Rqc1 is not cotranslationally degraded by the RQC complex.***A*, a TAP-tagged version of Rqc1 was used to measure the protein levels in the wt, *lnt1*Δ, *asc1*Δ, *rqc2*Δ, and *hel2*Δ strains. The band intensity was quantified and normalized in relation to the housekeeping protein phosphoglycerate kinase (Pgk1). *B*, qPCR analysis of *RQC1*-TAP mRNA levels in the wt, *lnt1*Δ, *asc1*Δ, *rqc2*Δ, and *hel2*Δ strains. The Rqc1 levels were normalized in relation to the housekeeping gene actin (*ACT1*). The analysis of differential expression was made by relative quantification using 2^−ΔΔ*Ct*^ method. One-way ANOVA with Bonferroni's multiple comparison test was used for statistical analyses. *C*, 28 to 32 and 21 to 23 nucleotide footprint analysis of *RQC1* shows neither accumulation of short reads at its polybasic site nor accumulation of long reads upstream of the stalling site (*left* and *right panels*, respectively). *Blue arrow* indicates the start of the polybasic sequences, and the *blue dotted line* indicates the hypothetical position of the collided ribosomes. The reads were plotted at an approximate position of the ribosomal A site. *D*, levels of the polybasic reporter GFP-R12-RFP subjected to 10 h cycloheximide (CHX) treatment at increasing concentrations. *E*, the same experiment as panel *D* was performed with an Rqc1-TAP strain.

To investigate the mechanism of Rqc1 regulation by the RQC, we analyzed Rqc1 expression in the backgrounds *ltn1*Δ, *asc1*Δ, *rqc2*Δ, and *hel2*Δ. We used a C-terminal TAP-tagged version of Rqc1, meaning that we can only observe an increase in the levels of the full-length peptide. As previously described, the deletion of *LTN1* increased the expression levels of Rqc1 but, different from the results reported in Brandman *et al.*, 2012, we observed that the deletion of *ASC1*, *HEL2*, or *RQC2* had no impact on the full-length Rqc1 expression levels (Fig. 5*A*). Rqc1 regulation was different from that of GFP-R12-RFP, an artificial reporter with a polybasic sequence known to be a target of the RQC (7, 21, 22, 24). As expected, the reporter presented increased levels of GFP (truncated peptide) when *LTN1* was deleted and increased levels of both GFP and RFP (full-length peptide) when *ASC1* or *HEL2* was deleted (Fig. S11). To test if the TAP-TAG had any influence in our results, we added it to the C-terminal of the construct GFP-R12-RFP (stalling) and GFP-ST6-RFP (nonstalling) (Fig. S12) and measured its expression in wt, *ltn1*Δ, *asc1*Δ, and *hel2*Δ cells (Fig. S13). Our results showed that the presence of the TAP-TAG did not alter the expression of the neither constructs (Fig. S13). The amount of TAP-TAG nonstalling reporter was similar for all strains, while higher levels of TAP-TAG stalling reporter were observed for *asc1*Δ and *hel2*Δ strains (Fig. S13).

It is noteworthy that the Rqc1 polybasic sequence (blue arrow) is located at the protein N-terminal, meaning that only the upstream polypeptide fragment should be stabilized in an *ltn1*Δ strain. Therefore, as previously mentioned, the overexpression of the full-length Rqc1 in the *ltn1*Δ strain (Fig. 5*A* and Brandman *et al.*, 2012) is inconsistent with the current model for RQC action. The *RQC1* mRNA levels were the same in all tested backgrounds (Fig. 5*B*). To test if the presence of the C-terminal TAP-TAG influenced *RQC1* mRNA levels, we repeated the qPCR analysis with the nontagged wt *RQC1*, and the same results were observed (Fig. S14). Moreover, ribosome profiling of *RQC1* did not show the characteristic footprint pattern of ribosome collisions and accumulation of short fragments (Fig. 5*C*—blue dashed lines, see also Fig. S15), further indicating that the Rqc1 polybasic sequence does not act as a strong stalling sequence.

Another way to verify whether a transcript is a target of the RQC complex is through the saturation of the RQC system; if the protein of interest is a target, the saturation of the RQC will increase its expression levels. The saturation can be accomplished by treatment with low concentrations of an elongation inhibitor such as CHX, which leads to multiple events of ribosome stalling and RQC activation (7, 27). Accordingly, Figure 5*D* shows that the GFP signal from the GFP-R12-RFP reporter increased at subsaturation CHX concentrations and that this effect was Ltn1-dependent (Fig. 5*D*). However, when we tested the Rqc1 TAP-TAG strain, we could not observe the same result, further indicating that the RQC complex does not control the final Rqc1 protein output during translation (Fig. 5*E*). In another experiment, yeast cells were treated with 50 μg/ml CHX, a concentration high enough to completely halt translational activity. We then followed the levels of the Rqc1-TAP protein over time. The half-life of Rqc1-TAP protein was much shorter in wt cells when compared with *lnt1*Δ cells (Fig. S16, *A*–*D*). This result suggests that Rqc1 decay rate depends on Ltn1, but is uncoupled from its translation process. This experiment provides further support to the model of a posttranslational regulation of Rqc1 by Ltn1 (Fig. S16*E*).

## Discussion

The hypothesis that the translation of positive amino acids leads to ribosome stalling and translation repression has been disputed since shortly after its proposal. The Grayhack group was the first to propose that codon bias was the major factor behind ribosome stalling observed with positive sequences (67, 70, 71, 72). They examined the influence of all sequences of 10 repeated codons on luciferase activity, and they demonstrated that the codon choice had a stronger effect than the final charge. Of particular interest is the codon CGA in *S. cerevisiae*, which leads to a 99% reduction in luciferase activity in comparison with the codon AGA, both coding for arginine (73). In recent works, they proposed that the CGA codon does not need to be highly concentrated to cause translational repression: the presence of as few as two consecutive CGA codons (68, 72) or specific combinations of CGA with a second codon, the so-called inhibitory pairs (57, 74), is sufficient to inhibit translation. In the same token, the Green and Djuranovic groups proposed that poly (A) sequences are the major cause of translation repression observed with polylysine stretches (12, 43, 53, 75). They showed that the presence of six AAA codons in tandem leads to an approximately 30% to 70% reduction in protein expression in comparison to AAG in different organisms and that this reduction is proportional to the quantity of AAA (12, 53). Letzring *et al.*, 2010, (73) had already observed that the translation of the codon AAA is worse than that of AAG, but what both the Green and the Djuranovic groups demonstrated is that the translation disruption is caused by the presence of a poly (A) sequence in the mRNA, independent of codon choice. Sequences of AAA codons followed or preceded by codons rich in adenine (preceded by CAA and followed by AAC, for example) can cause greater translation repression than the same sequences of AAA codons alone (12). The presence of an extended poly (A) sequence can cause ribosomal sliding, a slow ribosome frameshifting movement that leads to the incorporation of extra lysine residues in the nascent peptide. More importantly, it leads to the translation of premature termination codons, which are targeted by nonsense-mediated decay and lead to translation termination (53). This may be caused by the helical conformation that poly (A) sequences can adopt, which allows them to enter the ribosomal A site and inhibit translation (43).

On the other hand, there is still evidence that the charge itself can play a role in translation dynamics. Positively charged sequences, independent of codon choice, are translated at slower rates than negatively charged or noncharged sequences (44, 56) and are still considered a hallmark of ribosome stalling. Additionally, proteins with a high density of negatively charged amino acids in a 30-residue stretch are far more common than proteins with a high concentration of arginines and/or lysines in 30 residues, suggesting a negative selection pressure against long polybasic sequences (56, 76). Moreover, even in the papers that highlight the effect of codon choice on the inhibitory effect observed with polybasic sequences, we can see evidence that charge alone is still a relevant factor for translation efficiency. Both Arthur *et al.*, 2015, and Koutmou *et al.*, 2015, show that the translation of 12 AGA codons leads to a reduction of approximately 50% in protein amount (12, 53). Taken together, the literature suggests that the rare codons (CGA) and poly (A) sequences are detrimental for translation but does not rule out the effect of positively charged segments as RQC complex activation factors (77, 78, 79).

Our search for endogenous targets of the RQC complex was motivated by the possibility of translation repression caused by positively charged segments, as described above. Rqc1 was the first example of an endogenous protein with a proposed cotranslational regulation promoted by the RQC complex (7). Our results show that polybasic sequences similar to Rqc1 are relatively common (Fig. 1), and even though they are translated at slower rates (Fig. 3*A* and Fig. S2) (44), it seems that this is not sufficient to terminate translation and trap ribosomes in the empty A site, as characterized by the stabilization of 20 to 22 nucleotide footprints (39, 43) (Fig. 3*B*, Fig. S4). Consistent with our results that polybasic sequences do not cause premature termination, not even the most positively charged sequences could be identified as endogenous targets of the RQC complex under the conditions tested (Fig. 4). Additionally, our results corroborate recent findings showing that short polybasic sequences promote ribosome collisions and are enriched in the ribosome exit tunnel of the disomes (30). Recent results suggest that disomes are likely to resume translation without recruiting the RQC pathway and, they hypothesize that these polybasic-induced pauses (and collisions) can favor the cotranslational folding of upstream sequences (30). Recently, the yeast gene *SDD1* was identified as a natural target of RQC. The stalling sequence contains a rare combination of inhibitory features: a stretch of bulky amino acids, followed by a polybasic sequence with a CGA-CGA ICP and a short poly (A) tract. The result is a combination of amino acid−specific inactivation of the peptidyl-transferase center at the 60S subunit and mRNA-specific obstruction of the decoding center at the 40S subunit (26). It is important to note that mutations on amino acids upstream of the polybasic sequence were able to increase the levels of Sdd1 protein expression (26), revealing that the polybasic sequence of Sdd1 was necessary, but not sufficient, to cause translation repression. Agreeing with our data, it appears that, in most cases, the translation of polybasic sequences does not lead to severe ribosome stalling, but it can become troublesome when combined with additional characteristics. Future studies will help to identify and understand how these characteristics affect the mechanisms of cotranslational regulation.

Similar to what was observed with other proteins with polybasic sequences, with the exception of Sdd1, we showed that the RQC complex does not inhibit Rqc1 translation. In our hands, *rqc2*Δ, *hel2*Δ, and *asc1*Δ did not significantly affect Rqc1 levels, which speak against an RQC complex-dependent regulation (Fig. 5*A*). However, Rqc1 overexpression in an *ltn1*Δ strain agrees with the result from Brandman *et al.*, 2012, which, at the time, was interpreted as evidence that the Rqc1-Flag nascent protein was being targeted by the RQC complex. However, the deletion of *LTN1* alone is usually associated with the stabilization of translation intermediates (up to the stalling point), as we and others observed in the GFP-R12-RFP reporter (Fig. S11), not full-length constructs as observed with Rqc1-TAP. Taking our data into account, we speculate that the K-rich basic sequence can function as the ubiquitylation site involved in Rqc1 regulation. Since it was previously reported that Ltn1 could ubiquitylate substrates independently of the RQC complex (80, 81), we speculate that Ltn1 might regulate Rqc1 in a process that is uncoupled from Rqc1 translation (Fig. S16*E*).

## Experimental procedures

**Table:** 

Data	Source	Identifier
Translation efficiency	Weinberg *et al.*, 2016 (59)	SRR1049521
*S. cerevisiae* drug-free Riboseq data	Pop *et al.*, 2014 (38)	SRR1688545
*S. cerevisiae* + CHX + ANS Riboseq data	Wu *et al.*, 2019 (39)	SRR7241922
*S. cerevisiae* + CHX + TIG Riboseq data	Wu *et al.*, 2019 (39)	SRR7241919
*S. cerevisiae* + CHX Riboseq data wt #1	Matsuo *et al.*, 2020 (26)	SRR9054693
*S. cerevisiae* + CHX Riboseq data wt #2	Matsuo *et al.*, 2020 (26)	SRR9054694
*S. cerevisiae* + CHX Riboseq data *hel2Δ* #1	Matsuo *et al.*, 2020 (26)	SRR9054695
*S. cerevisiae* + CHX Riboseq data *hel2Δ* #2	Matsuo *et al.*, 2020 (26)	SRR9054696
*S. cerevisiae dom34Δ, ski2Δ* disome	D'Orazio *et al.*, 2019 (40)	SRR8830773
*S. cerevisiae* wt disome #1	Meydan and Guydosh, 2020 (41)	SRR10302104
*S. cerevisiae* wt disome #2	Meydan and Guydosh, 2020 (41)	SRR10302108
Kozak score 50 nt	Cuperus *et al.*, 2017 (62)	
Kozak score 5 nt	Cuperus *et al.*, 2017 (62)	
Codon stabilization coefficient gene (CSCg)	Carneiro *et al.*, 2019 (60)	
Time for translation initiation	Siwiak and Zielenkiewicz, 2010 (61)	
40S ribosomal small subunit (SSU) footprint profiling	Archer *et al.*, 2016 (63)	
Software and algorithms		
Net charge calculation	Requião *et al.*, 2017 (56)	
Poly (A) finder	This paper	https://github.com/mhoyerm/adenosine_sequence
Consecutive K and/or R finder	This paper	https://github.com/mhoyerm/arg_lys_sequence
Stretches of K and/or R finder	This paper	https://github.com/mhoyerm/arg_lys_stretches
A site estimation	This paper	https://github.com/RodolfoCarneiro/RPF-build

### Statistical analyses and raw data

The raw data used to create Figures 1 and 2, and the statistical analysis *t*-test calculations are presented in Tables S2 and S4. The Spearman correlation among the parameters used in Figure 2 is presented in Table S3. All statistical analyses were performed with GraphPad Prism 7.

### Data sources

Coding sequences and the annotation of *S. cerevisiae* were obtained from the *Saccharomyces* Genome Database (SGD; https://www.yeastgenome.org) and Ensembl Genomes (http://ensemblgenomes.org/). We excluded dubious ORFs as defined in the *Saccharomyces* Genome Database from our analysis. The list of inhibitory codon pairs (ICP) and genes with ICP is presented in Table S1.

### Net charge calculation

We developed a program that screens the primary sequence of a given protein and calculates the net charge in consecutive frames of a 30-amino-acid window, the approximate number of amino acids that fill the ribosome exit tunnel (55, 56). For the net charge determination, K and R were considered +1; D and E were considered −1; and every other residue was considered 0. The additional charges of the free amino and carboxyl groups of the first and last amino acids, respectively, were disregarded. In a previous publication, we have shown that these simplified parameters are equivalent to a calculation using partial charges of individual amino acids at pH 7.4, according to their p*K*a values and the Henderson–Hasselbalch equation (56). The algorithm initially establishes a stretch containing a predetermined number of amino acids (#1 to #30, for example). The stretch charge is calculated, and the charge value and the position of the first amino acid are temporarily saved to the memory. Then, our algorithm advances to the next stretch, from amino acid #2 to amino acid #31, and performs the same analysis until the end of the protein.

### Codon stabilization coefficient (CSC) calculation

Based on Pearson’s correlation between the frequency of occurrence of each codon in each mRNA and the mRNA half-lives, Coller and colleagues created the codon occurrence to mRNA stability coefficient (CSC) (82). We designed and implemented an algorithm that calculated the mean value of the CSC for each gene (CSCg) (60).

### Poly (A) finder

An algorithm was developed to locate the position of stretches containing only adenine (A) in an ORF. The algorithm finds adenine stretches of a chosen size for each ORF and generates as output a file containing the gene name, the stretch size, and its position in the ORF. For our analysis, we considered stretches of size equal to or larger than ten adenines.

### Stretches of K and/or R finder

We developed an algorithm that searches for stretches containing a certain number of arginines and/or lysines in a stretch of ten amino acids in a protein. The chosen number of K and/or R in the stretches was equal to or greater than 8. The input file of the algorithm was a proteome fasta file, and the output file returned the protein name and the position of the stretch found for each protein.

### Consecutive K and/or R finder

An algorithm was developed to find the location of stretches containing consecutive arginines and/or lysines in a protein. The stretches analyzed had a size equal to or larger than 6. The input file of the algorithm was a proteome fasta file, and the output file returned the protein name, the stretch size, and the position of the stretch in the protein.

### Yeast strains and growth conditions

Since no commercial antibodies are available for the proteins used herein, we used a TAP-TAG collection of yeast (65). In this collection, each ORF was tagged with a C-terminal TAG, and the same antibody could be used to detect the full collection. For each potential RQC complex target analyzed, a knockout strain for the *LTN1* or *ASC1* gene was created by homologous recombination. For the Rqc1 TAP-TAG strain, knockout strains for *RQC2* and *HEL2* genes were also created. After the creation of the knockout strains, the level of the TAP-tagged proteins was compared with that of the wild-type strain by western blot analyses. The TAP-TAG collection strains were cultivated in YPD medium (1% yeast extract, 2% peptone, 2% glucose). The *S. cerevisiae* BY4741 strain transformed with GFP-R12-RFP vector (R12: CGG CGA CGA CGG CGA CGC CGA CGA CGA CGG CGC CGC) (7) was cultivated in minimum synthetic medium, uracil dropout (0.67% Difco Yeast Nitrogen Base without amino acids, 2% glucose, and 0.003% methionine, leucine, and histidine).

### Yeast strain constructions

For knockout creation, *S. cerevisiae* BY4741 yeast (TAP-TAG collection) was transformed using the PEG/Lithium Acetate method (83). The PCR products used in the transformation were obtained by the amplification of the KanMX6 gene contained in the pFA6a-KanMx6 vector. All genes were knocked out by the insertion of the geneticin resistance gene into their original ORFs. The knockouts were confirmed using four sets of PCR. Two PCR sets combined primers for the target gene flank region (5’UTR or 3’UTR) with resistance gene primers. The other two PCR sets combined primers for the target gene flank region and ORF gene target primers. The primers used for deletions, strains construction, and confirmation are depicted in the topic “List of primers and PCR condition.” The *S. cerevisiae* BY4741 strains transformed with GFP-R12-RFP-TAP-TAG or GFP-ST6-RFP-TAP-TAG vectors (ST6: UCU ACU UCA ACA UCU ACA UCA ACU UCU ACC UCA ACG) were cultivated in minimum synthetic medium, uracil, and histidine dropouts.

### List of primers and PCR condition

**Table:** 

Primer	Sequence 5’-3’	Annealing temperature (T)/extension time (time)
Ltn1-F-deletion	TAAGCCATCAAAAAAAGTTCAAGCAATAGTTGGTTCTTAATGCGTACGCTGCAGGTCGAC	T = 54 °C/Time = 3 min
Ltn1-R-deletion	AAATGTCGTACATTTATATGAAATTTATATGCGATAGTCTAATCGATGAATTCGAGCTCG	
Ltn1F1	GGAGAGGTCTCCGGTTCGATTCC	T = 60 °C/Time = 1 min
KanB	CTGCAGCGAGGAGCCGTAAT	
Ltn1F1	GGAGAGGTCTCCGGTTCGATTCC	T = 56 °C/Time = 1 min
Ltn1R1	CAGTTTAGCATATATCTGCGACCAACA	
Ltn1C	TAACAAATATCCAAGTTAATGGCGT	T = 54 °C/Time = 1 min
Ltn1D	GAACAATTGAGAGAATGAAAAAGGA	
KanF1	TATGGAACTGCCTCGGTGAG	T = 54 °C/Time = 1 min
Ltn1D	GAACAATTGAGAGAATGAAAAAGGA	
Asc1-F-deletion	CCAAAAAATCCTTATAACACACTAAAGTAAATAAAGTGAAAAATGCGTACGCTGCAGGTC	T = 54 °C/Time = 3 min
Asc1-R-deletion	CTAGAAGATACATAAAAGAACAAATGAACTTTATACATATTCTTAATCGATGAATTCGAG	
Asc1F1	CCTGGCCATCTGTAGCCTTA	T = 54 °C/Time = 1 min
KanB	CTGCAGCGAGGAGCCGTAAT	
Asc1F1	CCTGGCCATCTGTAGCCTTA	T = 54 °C/Time = 1 min
Asc1R1	TGGCAACATCCCATAATCTCAAGG	
Asc1F2	GGACGGTGAAATTATGTTGTGG	T = 54 °C/Time = 1 min
Asc1R2	CGCAGCAAACAGAAAGCATAG	
KanF1	TATGGAACTGCCTCGGTGAG	T = 54 °C/Time = 1 min
Asc1R2	CGCAGCAAACAGAAAGCATAG	
Hel2-F-deletion	TCGAAAAAATAGTGGCTATACTTCTTTTGAAGAATTAGGATGCGTACGCTGCAGGTCGAC	T = 54 °C/Time = 3 min
Hel2-R-deletion	ATGCTATTGTCAGTTACAGGTTAGAAATATATTTCCAACTAATCGATGAATTCGAGCTCG	
Hel2A	AGTGACCTCGTTATACATATCCCTG	T = 54 °C/Time = 1 min
KanB	CTGCAGCGAGGAGCCGTAAT	
Hel2A	AGTGACCTCGTTATACATATCCCTG	T = 54 °C/Time = 1 min
Hel2B	AAAGTCACAAACTTGTTCATCCTTC	
Hel2C	TTGAAAAGTCTCAACCTACCTCAAC	T = 54 °C/Time = 1 min
Hel2D	ATTTTGTTGGAGTTGTCTTATGAGC	
Rqc2-F-deletion	TTATCCGGTCTAAGAAGTCAGGCAGGCAAGAGATTAATAGCGATGCGTACGCTGCAGGTCGAC	T = 54 °C/Time = 3 min
Rqc2-R-deletion	AAAATTATAATTGCTGTCTATTTTCTTTTCATCTCATATGATTTATCGATGAATTCGAGCTCG	
Rqc2A	TCTGTGGTTACGATTAAAATTGGAT	T = 54 °C/Time = 1 min
KanB	CTGCAGCGAGGAGCCGTAAT	
Rqc2A	TCTGTGGTTACGATTAAAATTGGAT	T = 54 °C/Time = 1 min
Rqc2B	CAATCGACAACAACATTGAGTTTAG	
Rqc2C	ACAAGTTAAAAGTAACAATCGCTGG	T = 54 °C/Time = 1 min
Rqc2D	ATTGATAATGGTGTTATCCTCGAAA	
KanF1	TATGGAACTGCCTCGGTGAG	T = 54 °C/Time = 1 min
Rqc2D	ATTGATAATGGTGTTATCCTCGAAA	
TAP-TAG-F-BoxI	NNNNNNNGACCTACGTCGGTCGACGGATCCCCGGGTT	T = 65 °C/Time = 3 min
TAP-TAG-R-NotI	ATAAGAATGCGGCCGCTCGATGAATTCGAGCTCGTT	
RFP-F	GATCAGAGGGGTGAACTTCCC	T = 55 °C/Time = 1 min
TAP-TAG-R	CTTCATCGTGTTGCGCAAGG	
RFP-F	GATCAGAGGGGTGAACTTCCC	T = 55 °C/Time = 2 min
Plasmid-R	CCAGAAAGCATTCATCGCGT	

### Protein extraction and SDS-PAGE

The protein extraction of the strains was conducted in the exponential phase of growth (O.D ∼ 0.6–0.8), treating equal quantities of cells using the NaOH/TCA method (84). For the SDS-PAGE, equal sample volumes were applied on the acrylamide gels and submitted to an electrophoretic run. For the YHR131C TAP-TAG strain, protein extraction was conducted using a glass bead protein extraction protocol (85). Total protein quantification of the glass bead protein extracts was conducted using the BCA Protein Assay kit (Thermo Fisher), and samples with equal amount of proteins were applied on SDS-PAGE gels. The acrylamide gel concentration changed between 10 and 12.5% according to the size of the analyzed protein.

### Western blotting

Proteins separated by SDS-PAGE were transferred to PVDF membranes using the TRANS-BLOT semidry system from BIO-RAD or the wet blotting system in transfer buffer (25 mM Tris, 192 mM glycine, 0.1% SDS, 20% methanol, pH 8.3). The membranes were blocked using a Li-Cor blocking buffer with an overnight incubation at room temperature. Incubation with primary antibodies was performed at 4 °C for at least 1 h. The membranes were incubated in Li-Cor Odyssey secondary antibodies at room temperature for at least 1 h and then revealed in an Odyssey scanner. The primary antibodies used were mouse anti-PGK1 (Invitrogen; dilution 1:10,000), rabbit anti-TAP-TAG (Thermo Scientific; dilution 1:10,000), mouse anti-GFP (Sigma; dilution 1:2000), and rabbit anti-Asc1 (dilution 1:5000) (86). The secondary antibodies that were used were anti-mouse 680 LT Li-Cor (dilution 1:10,000) and anti-rabbit 800 CW Li-Cor (dilution 1:10,000).

### qRT-PCR

For qRT-PCR analysis, the Rqc1 TAP-TAG knockout strains constructed in this work were cultivated until the exponential phase (O.D ∼ 0.6–0.8) and RNA from these cells were extracted following the hot phenol method. The same protocol was used for the corresponding knockouts from a commercial collection as a control. After extraction, the RNAs were submitted to DNase I (Thermo Scientific) treatment for DNA contamination elimination. The reverse transcription was performed using random primers with the High-Capacity cDNA Reverse Transcription Kit (Thermo Scientific). For the cDNA amplification, we used SYBR Green PCR Master Mix on the StepOne Plus System (Applied Biosystems). The following protocol was used on the thermocycler: 95 °C for 10 min and 40 cycles of 95 °C for 15 s and 60 °C for 1 min, melting curve analysis: 95 °C for 15 s, 60 °C for 1 min and 95 °C for 15 s. The primers used were: 5’CGGAATGCACCCGCAACATT and 5’CGGCCTTTCCCATAATTGCCA for *RQC1*, and 5’ TTCCCAGGTATTGCCGAAA and 5’ TTGTGGTGAACGATAGATGGA for *ACT1*. The analysis of differential expression was made by relative quantification using 2^−ΔΔ^Ct method, and the statistical analysis was conducted on Prism.

### TAP-TAG cloning in GFP-R12-RFP and GFP-ST6-RFP plasmids

First, the TAP-TAG sequence was amplified from the genome of a TAP-TAG collection strain using primers with restriction sites inserted on the extremities of the sequence. The primers were designed to amplify the TAP-TAG sequence and the His3Mx6 marker. The PCR product was cleaned using the Wizard SV Gel and PCR Clean-Up System (Promega) and the purified PCR was used in a double digestion reaction with Box I (Thermo Scientific) and Not I (Fermentas) restriction enzymes. The double digestion reaction was conducted in buffer Tango 1X in an overnight incubation at 37 °C. The same double digestion reaction was conducted with the plasmids (GFP-R12-RFP and GFP-ST6-RFP) (21). Both PCR and plasmids digested were purified with Wizard SV Gel and PCR Clean-Up System (Promega). The ligation reaction was done using the 3:1 proportion of vector to insert, where 150 ng of vector and 126 ng of insert were used. The ligation reaction with the T4 DNA ligase (Invitrogen) was conducted following the manufacturer’s instructions. *Escherichia coli* DH5α competent cells were used to receive the plasmids after the ligation reaction was completed. To confirm the TAP-TAG insertion in the colonies two sets of colonies PCR were done. In the first one, we observed TAP-TAG presence in the 3’ extremity of RFP sequence. In the second set we observed the sequence of TAP plus His3Mx6 marker in the 3’ extremity of RFP sequence. The cloning strategy removes 63 nt of the RFP sequence for TAP-TAG sequence insertion. Another confirmation was done by western blot, where we observed an increase in the full-length protein by approximately 28 kDa, the size of the TAP-TAG insertion. Moreover, just strains transformed with the TAP-TAG plasmids presented immunoreactivity against the TAP-TAG antibody (Fig. S13). The primers used for both to amplify the TAP-TAG sequence from the genome and for the confirmation steps are depicted in the topic “List of primers and PCR condition.”

### Cycloheximide (CHX) treatment

For the experiments using serial dilutions of CHX, concentrations ranging from 10^−6^ to 10^−1^ mg/ml were used, with steps of ten times the dilution factor. Strains grew until the exponential phase and were treated with CHX for 10 h under shaking at 28 °C. For the pulse-chase experiment a concentration of 50 μg/ml of CHX was used. Strains also grew until the exponential phase and were treated with CHX under shaking at 28 °C. The points were removed in intervals of 20 min of incubation with the drug. Alternatively, points were also removed in intervals of 30 min after 10 h of incubation with CHX. The points collected were submitted to the protein extraction protocol.

### Ribosome profiling data

*S. cerevisiae* ribosome profiling data were treated as described previously (44, 56). The data were analyzed as described by Ingolia and collaborators (42), except that the program used here was Geneious R11 (Biomatter Ltd) instead of the CASAVA 1.8 pipeline. The data were downloaded from GEO (38, 39), and the adaptor sequence was trimmed. The trimmed FASTA sequences were aligned to *S. cerevisiae* ribosomal and noncoding RNA sequences to remove rRNA reads. The unaligned reads were aligned to the *S. cerevisiae* S288C genome deposited in the *Saccharomyces* Genome Database. First, we removed any reads that mapped to multiple locations. Then, the reads were aligned to the *S. cerevisiae* coding sequence database, allowing two mismatches per read. For 27 to 29 nt footprint analyses, the ribosome profiling data were obtained without any drug, and just fragments with 27 to 29 nt were used (38). For 20 to 22 nt footprint analyses, the ribosome profiling data were obtained with cells lysed with buffer containing 0.1 mg/ml of CHX and tigecycline (TIG) or 0.1 mg/ml of CHX and anisomycin (ANS) (39). We normalized the coverage within the same transcript by dividing each nucleotide read by the sum of the number of reads for each gene in a given window. A window of 150 nucleotides before and after the beginning of a stretch of interest was used to calculate the number of reads of each gene. For Figures 3, *C*–*G*, 5C, and Fig. S2, the ribosome profiling analysis was done following as described by Ingolia and collaborators (42). The data sets used in our analyses were from Pop *et al.*, 2014 (38) (GEO accession GSE63789) and from Matsuo *et al.*, 2020 (26) (GEO accession GSE131214). The data were mapped to the R64.2.1 S288C Coding Sequence (CDS) from the *Saccharomyces* Genome Database Project. The alignment was done using Bowtie accepting two mismatches at most. The analysis was restricted to 28 to 32 nt reads for Fig. S2. The A-site was estimated based on the read length: it was defined as the codon containing the 16th nt for 28 nt reads; the 17th nt for 29, 30, and 31 nt reads; and the 18th nt for 32 nt reads, considering the first nt as 1. The A-site estimation and other Ribosome Profiling Analysis were performed by custom software written in Python 3.8.2. For Figs. S2 and S3, we compared the ribosome profiles normalized for genes of the five distinct groups (Fig. S2) or all polybasic group (Fig. S3) with 2000 aleatory genes. Statistical analyses were performed with multiple *t*-tests using the Holm–Sidak method, and the adjusted *p*-value was calculated for each point (GraphPad Prism 7). For Figure 4, the ribosome profiling of each gene was downloaded from Trips-Viz by aggregating all experiments in the database (87). Disome Profiling Data: The data sets we used for disome profiling were from D’Orazio *et al.*, 2019 (40) (GEO accession GSE129128), and Meydan and Guydosh, 2020 (41) (GEO accession GSE139036). For the Meydan and Guydosh data, we further trimmed two nucleotides from the 5’ end of each read (the ones with the lowest quality in every read) and also five nucleotides from the 3’ end (adapting for the postmapping trimmings done to the nonmapped reads, as described in Meydan and Guydosh, 2020). Noncoding RNA sequences were removed from both data sets using Geneious. Then they were also mapped to the R64.2.1 S288C Coding Sequence (CDS), using Bowtie, accepting two mismatches at most. For D’Orazio data, we accepted any read containing between 50 nt and 80 nt. We estimated the A site from the first ribosome (the one at the 3’ portion of the read) as the codon containing the 46th nucleotide. For Meydan and Guydosh data, we accepted reads containing between 57 nt and 63 nt. Also, we estimated the A site from the ribosome at the 3’ portion of the read as the codon containing the 46th nucleotide.

### Read length distribution

For all data sets of ribosome profiling used herein, the read length distribution was calculated alongside the A-site prediction, using our own algorithm described in the Materials and Methods table.

### Clustering analysis

The clustering analysis of Figure 2*C* was performed by the Euclidean distance using Orange 3 software (88).

### Translation initiation normalized score

For Figure 2*C*, the genome was divided into eight equal groups based on their absolute values, from highest to lowest. The groups with the highest and the lowest values were further divided, making a total of ten groups. Each group was normalized individually. Their normalized values were then adjusted to fit the whole genome, meaning that the percentages from the group with the lowest values went from 0 to 100% to 0 to 6.25%, and the percentages from the group with the highest values from 0 to 100% to 93.75 to 100%, for example. This methodology was used to avoid the influence of a few genes with extremely high and low values that can make the visualization of the difference between the values of most proteins difficult.

## Data availability

The algorithms used in this work are available in the GitHub repository (https://github.com/mhoyerm).

## Supporting information

This article contains supporting information (7, 40, 41).

## Conflict of interest

The authors declare that they have no conflicts of interest with the contents of this article.
