# Supplementary material for: Rqc1 and other yeast proteins containing highly positively charged sequences are not targets of the RQC complex
Source: J Biol Chem. 2021 Mar 24;296:100586. doi: 10.1016/j.jbc.2021.100586 (PMC8102910; doi:10.1016/j.jbc.2021.100586)
Supplement: Supplemental Figures S1–S16 [file mmc5.pdf]

Support Information for:

Rqc1 and other yeast proteins containing highly positively charged sequences are not targets of the RQC complex

Géssica C. Barros[1#], Rodrigo D. Requião[1#], Rodolfo L. Carneiro[1#], Claudio A. Masuda[2], Mariana H. Moreira[1], Silvana Rossetto[3], Tatiana Domitrovic[4*] and Fernando L. Palhano[1*]

[1] Programa de Biologia Estrutural, Instituto de Bioquímica Médica Leopoldo de Meis, Universidade Federal do Rio de Janeiro, Rio de Janeiro, RJ, 21941-902, Brazil.

[2] Programa de Biologia Molecular e Biotecnologia, Instituto de Bioquímica Médica Leopoldo de Meis, Universidade Federal do Rio de Janeiro, Rio de Janeiro, RJ, 21941-902, Brazil.

[3] Departamento de Ciência da Computação, Universidade Federal do Rio de Janeiro, Rio de Janeiro, RJ, 21941-902, Brazil.

[4] Departamento de Virologia, Instituto de Microbiologia Paulo de Góes, Universidade Federal do Rio de Janeiro, Rio de Janeiro, 21941-902, Brazil.

* To whom correspondence should be addressed: Tel.: +55 21 3938-6761; Email: palhano@bioqmed.ufrj.br or domitrovic@micro.ufrj.br

# These authors contributed equally.

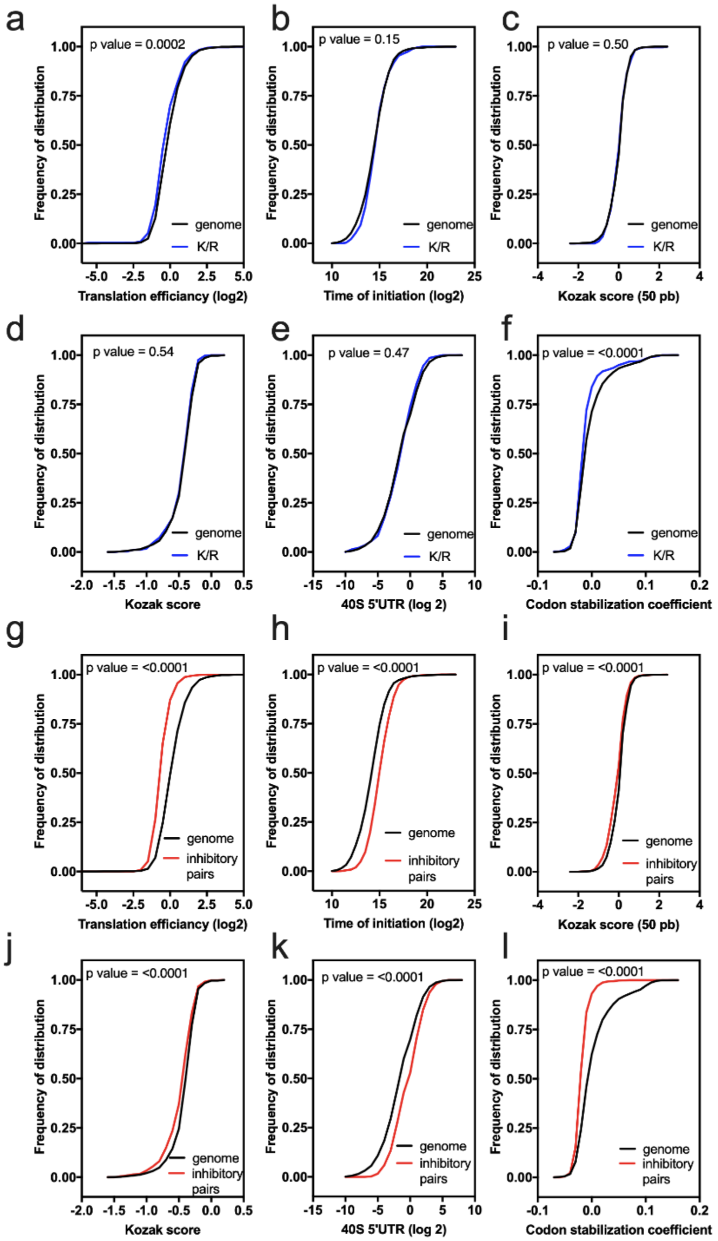

Figure S1. Translation initiation rate of genes with polybasic sequences. (a) The different groups of genes with some polybasic sequence were grouped in one single group (K/R) and compared regarding parameters that, to some extent, reflect translation initiation rate, with the genome (a-f). Kolmogorov-Smirnov test p values are showed for each comparison. As control was used, genes with one of the 17 inhibitory codon pairs (ICP) (g-l). The parameters used to infer initiation gene rate were; translation efficiency, time for translation initiation, Kozak score of the 50 first nucleotide, Kozak score of the first 5 nucleotide, 40S ribosomal small subunit (SSU) footprint profiling (SSU 5'UTR: start codon) and codon stabilization coefficient.

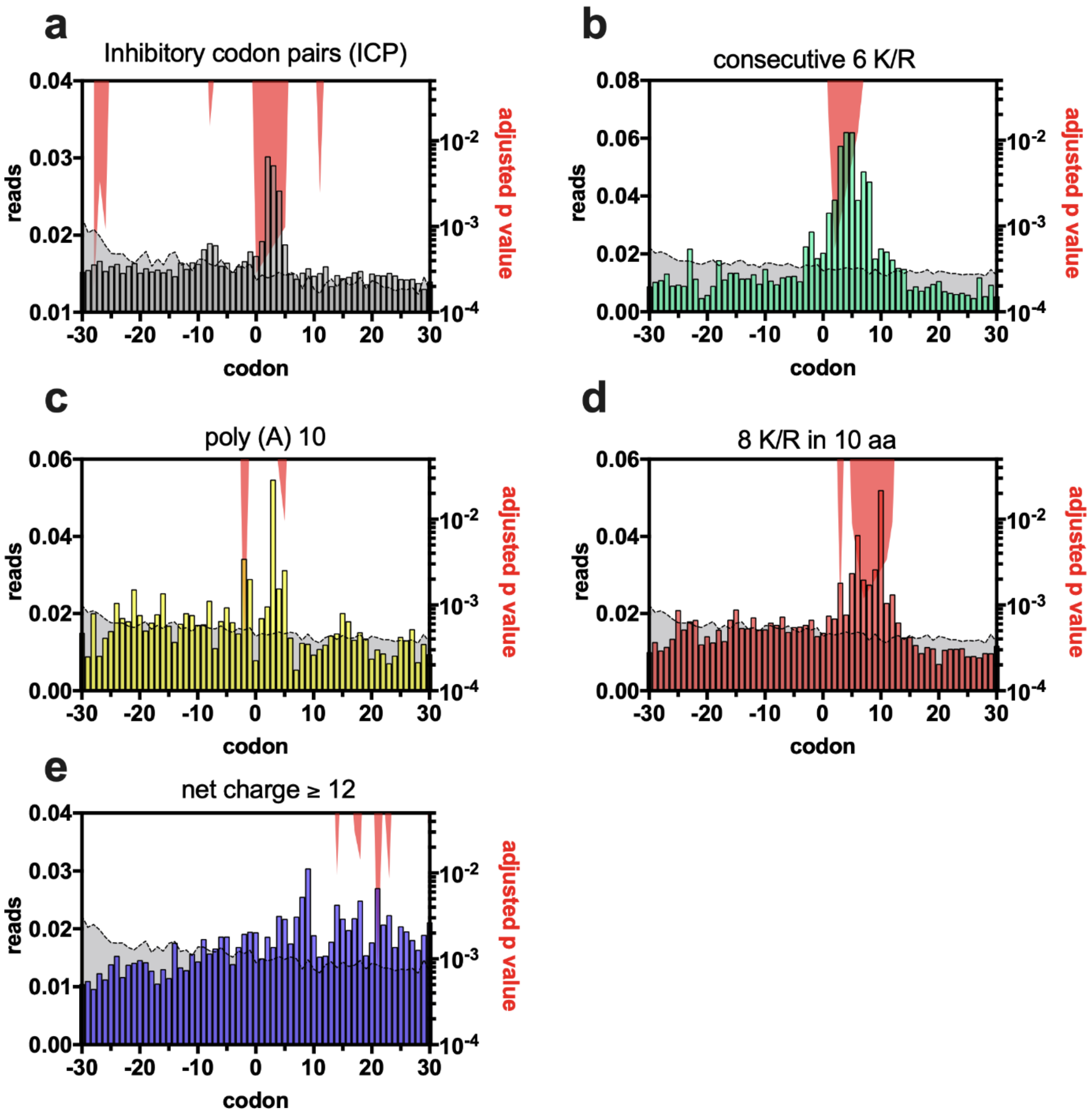

Figure S2. A site ribosome profiling of genes with poly-basic sequences. Ribosome profiling reads of the A site indicate that all poly-basic sequences are enriched with 28-32 nucleotide long footprints (b-e). As control, genes with one of the 17 inhibitory codon pairs (ICP) were used (a). The gray area represents the reads of a list of 2,000 random genes that allowed us to perform statistical analyses (multiple t-test using Holm-Sidak method, see details on methodology section). The right y-axis represents the adjusted p-value (red area).

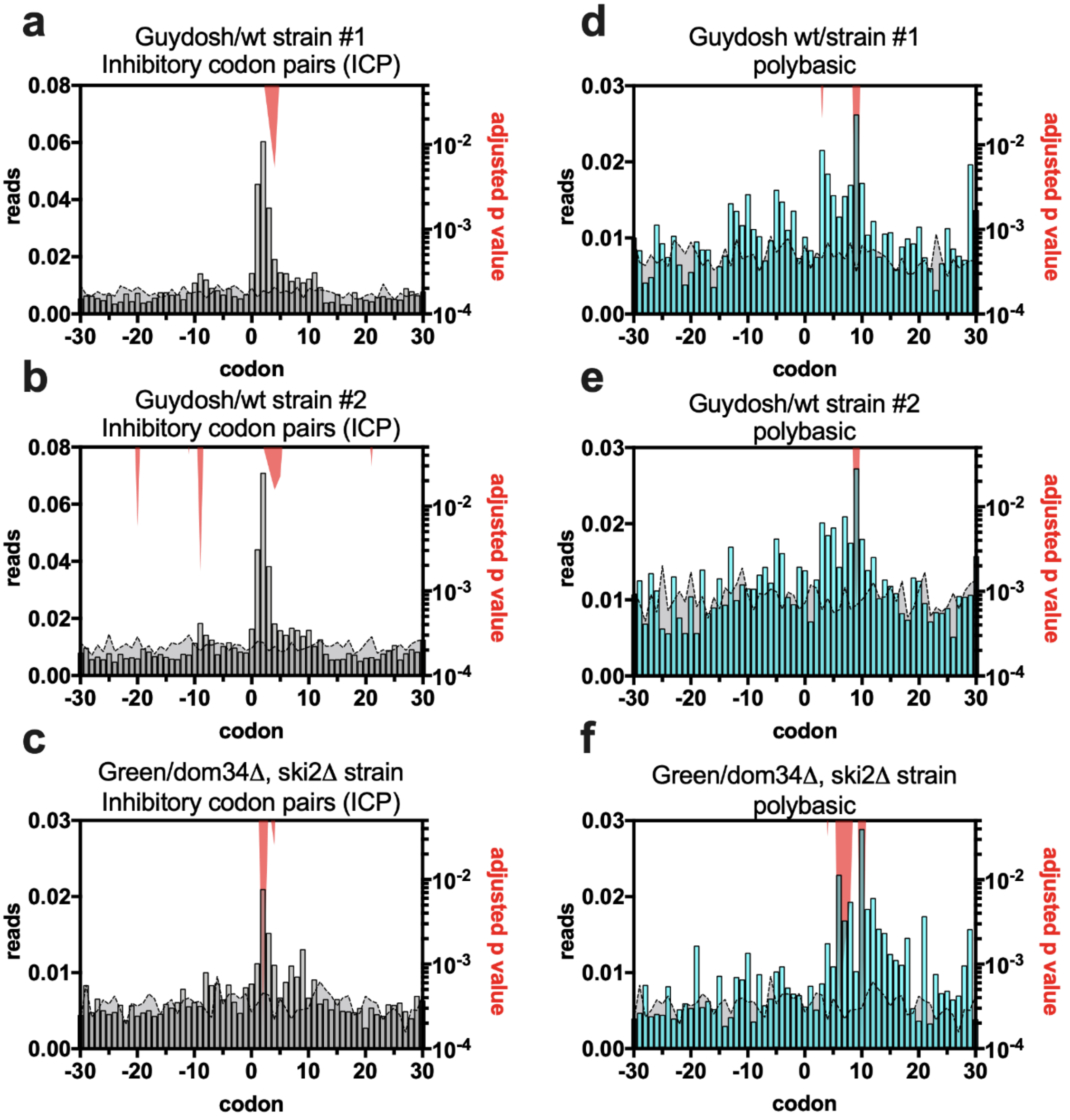

Figure S3. Disome profiling of genes with poly-basic sequences. Disome profiling reads of the A site indicate that genes with poly-basic sequences are enriched with disomes (d-f). As control, genes with one of the 17 inhibitory codon pairs (ICP) were used (a-c). The gray area represents the reads of a list of 2,000 random genes that allowed us to perform statistical analyses (multiple t-test using Holm-Sidak method, see details on methodology section). The right y-axis represents the adjusted p-value (red area). Three different disome datasets were used, two profiling's (#1 and #2) from a wild type strain (Guydosh: Meydam and Guydosh, 2020) and one profiling from a *dom34Δ, ski2Δ* strain (Green: D'Orazio et al, 2019). Due to the low ribosome profiling coverage, we grouped the four categories of polybasic sequences into one group namely, polybasic.

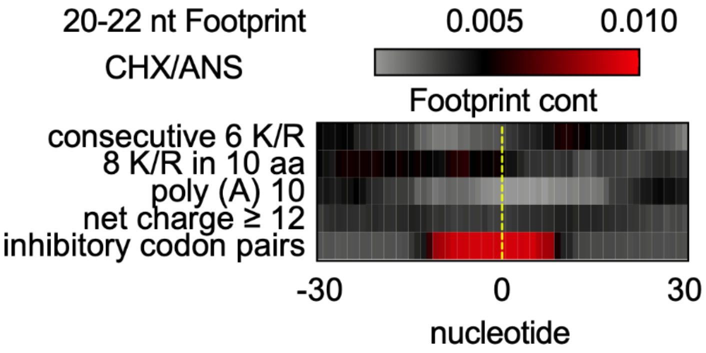

Figure S4. Ribosome footprint profiling of the genes with polybasic sequences from yeast genome. 20-22 nucleotide footprint analyzes of genes containing polybasic sequences. The dotted yellow line represents the start of the feature analyzed. As control was used, genes with one of the 17 inhibitory codon pairs (ICP). The 20-22 nt footprint was obtained by the use of cycloheximide and anisomycin (CHX/ANS).

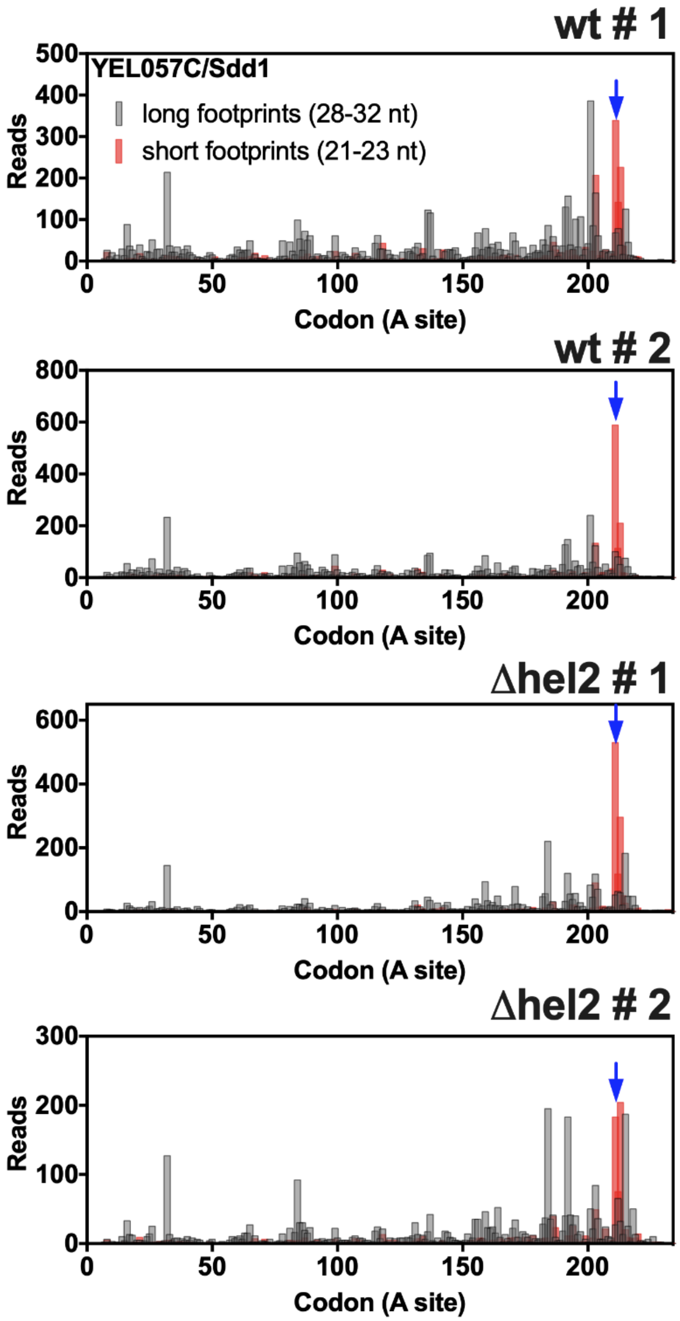

Figure S5. Ribosome profiling data of *SDD1*. 28-32 and 21-23 nucleotide footprint analysis of *SDD1* shows an accumulation of short reads at its polybasic site, indicating ribosome stalling, and accumulation of long reads upstream of the stalling site with a periodicity of roughly 10 codons, indicating ribosome collisions. The ribosome stalling is more evident in the *hel2*Δ strain. Blue arrow indicates the start of the polybasic sequences. The reads were plotted at an approximate position of the ribosomal A site. The two upper panels represent ribosome profiling obtained with a wt strain while the two in the bottom were obtained with a *hel2*Δ strain.

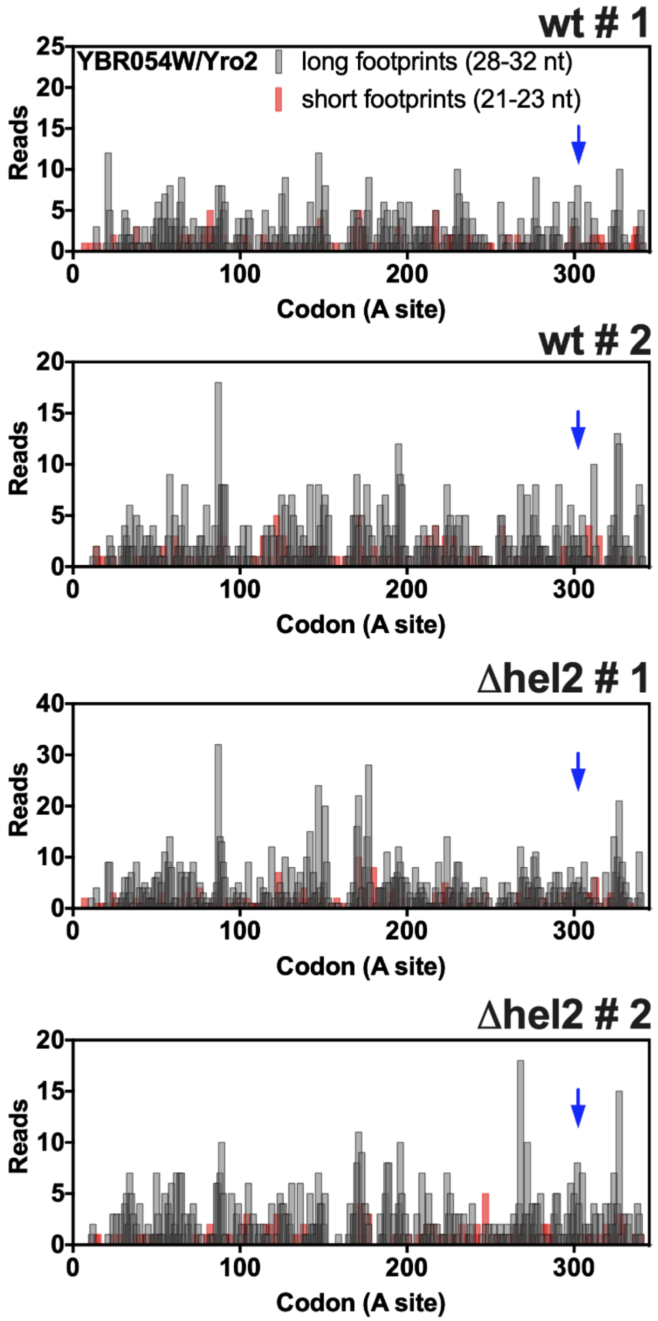

Figure S6. Ribosome profiling data of *YRO2*. 28-32 and 21-23 nucleotide footprint analysis of *YRO2*. Blue arrow indicates the start of the polybasic sequences. The reads were plotted at an approximate position of the ribosomal A site. The two upper panels represent ribosome profiling obtained with a wt strain while the two in the bottom were obtained with a *hel2*Δ strain.

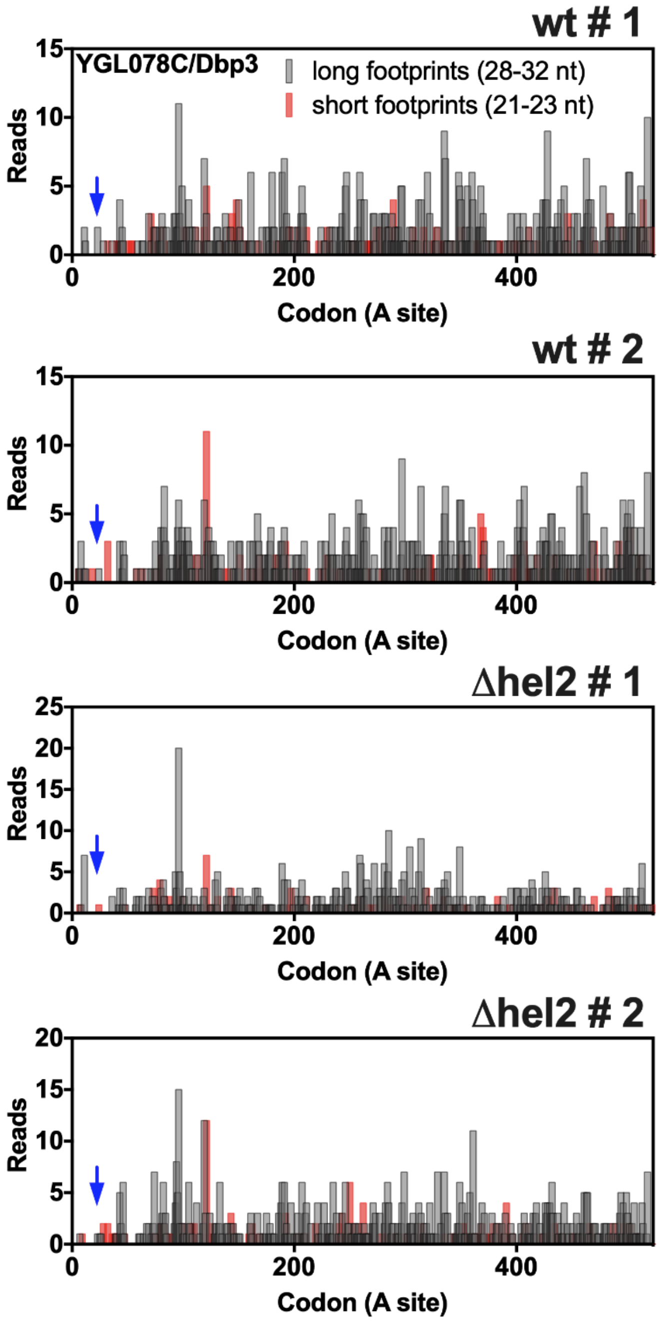

Figure S7. Ribosome profiling data of *DBP3*. 28-32 and 21-23 nucleotide footprint analysis of *DBP3*. Blue arrow indicates the start of the polybasic sequences. The reads were plotted at an approximate position of the ribosomal A site. The two upper panels represent ribosome profiling obtained with a wt strain while the two in the bottom were obtained with a *hel2*Δ strain.

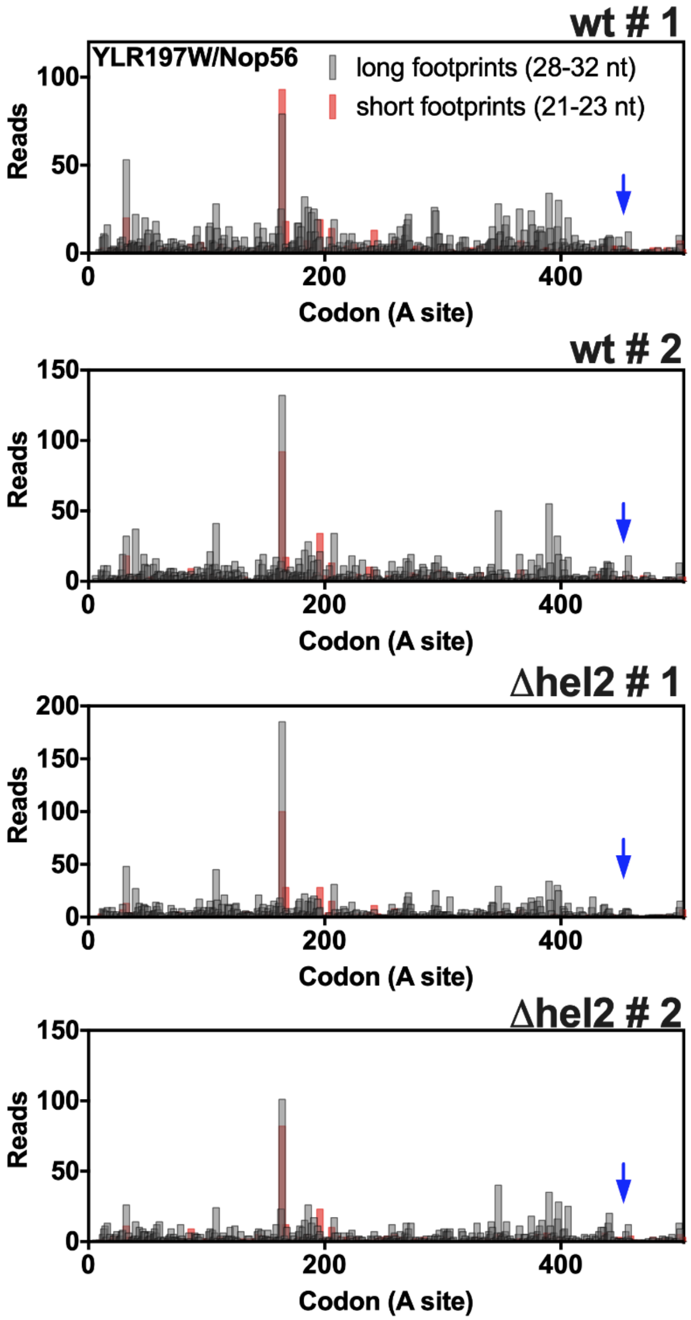

Figure S8. Ribosome profiling data of *NOP56*. 28-32 and 21-23 nucleotide footprint analysis of *NOP56*. Blue arrow indicates the start of the polybasic sequences. The reads were plotted at an approximate position of the ribosomal A site. The two upper panels represent ribosome profiling obtained with a wt strain while the two in the bottom were obtained with a *hel2*Δ strain.

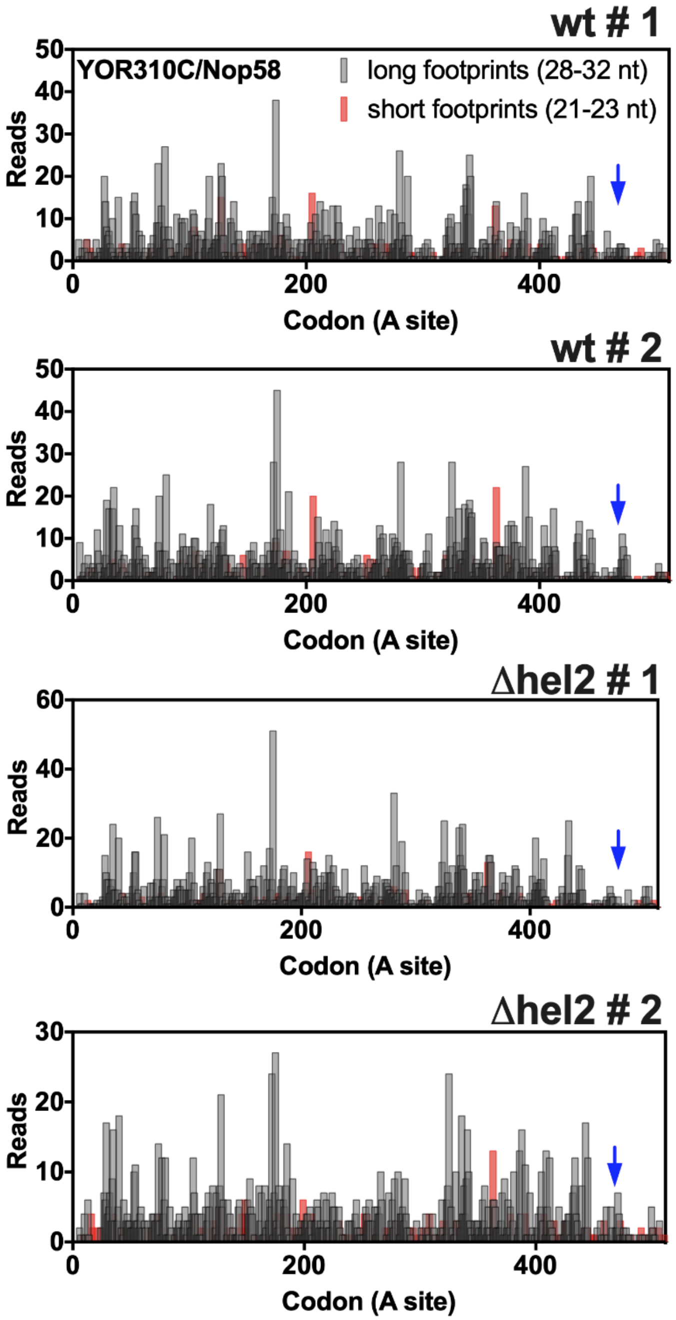

Figure S9. Ribosome profiling data of *NOP58*. 28-32 and 21-23 nucleotide footprint analysis of *NOP58*. Blue arrow indicates the start of the polybasic sequences. The reads were plotted at an approximate position of the ribosomal A site. The two upper panels represent ribosome profiling obtained with a wt strain while the two in the bottom were obtained with a *hel2*Δ strain.

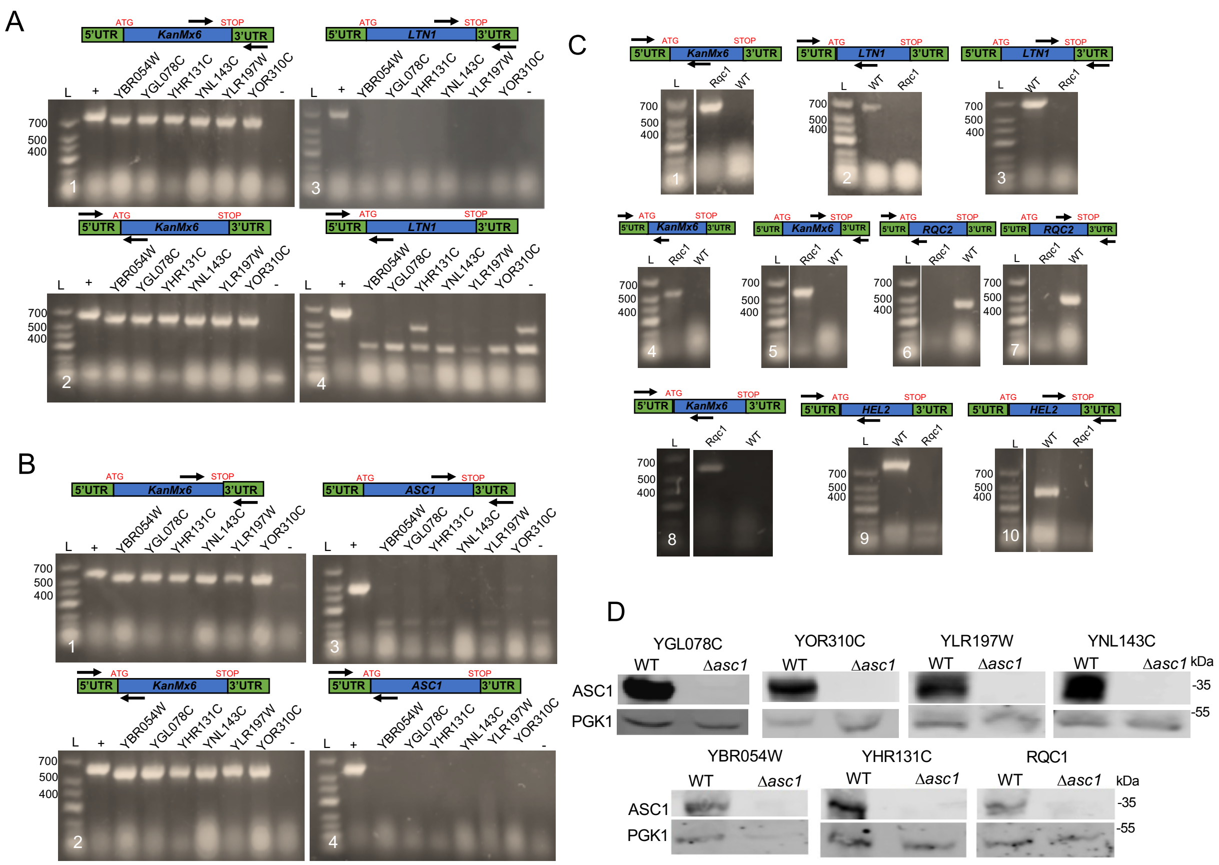

Figure S10. Confirmation of deletions in the select TAP-TAG protein targets and Rqc1 TAP-TAG. The gels showed in the panels a and b confirm the deletion of *LTN1* and *ASC1*, respectively, in all select targets. The four PCR sets show the presence of the *KanMx6* gene in the original gene ORF (gels 1 and 2), and the absence of *LTN1* (a) or *ASC1* (b) genes on their ORFs (gels 3 and 4). The DNAs from wt, *ltn1*Δ (knockout collection) and *asc1*Δ (knockout collection) strains were used as positive (+) or negative (-) controls of all the PCRs. (c) Deletions of *LTN1*, *RQC2* and *HEL2* were confirmed by PCR in the Rqc1 TAP-TAG strain. For *LTN1* deletion, gel 1 shows the presence of *KanMx6* gene and gels 2 and 3 the absence of *LTN1* on its original ORF. For *RQC2* deletion, gels 4 and 5 show the presence of *KanMx6* gene and gels 6 and 7 the absence of *RQC2* on its original ORF. For *HEL2* deletion, gel 8 shows the presence of *KanMx6* gene and gels 9 and 10 the absence of *HEL2* on its original ORF. (d) Deletion of Asc1 in all targets and Rqc1 was confirmed by Western Blot using an anti-Asc1 antibody. L= ladder.

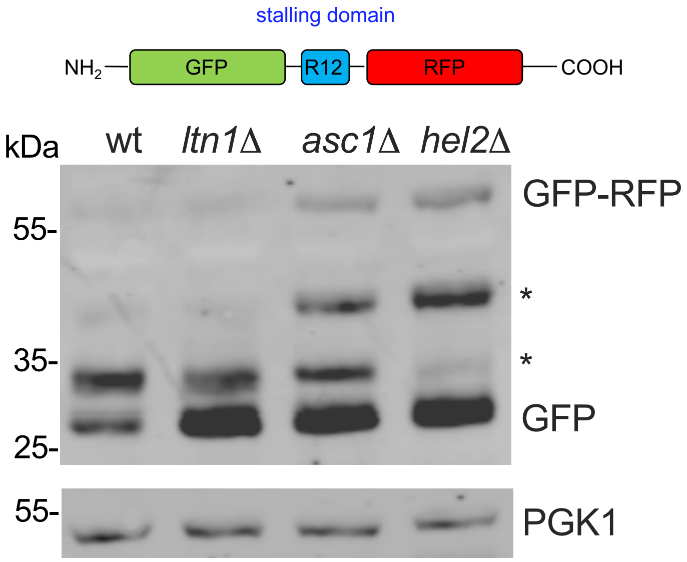

Figure S11. Whole-cell immunoblots of the indicated yeast strains expressing the stalling reporter (GFP-R12-RFP). GFP-RFP indicates the full-length translation product, and GFP indicates the arrest product. Stars denote products of the stalling reporter that likely to result from proteolytic cleavage.

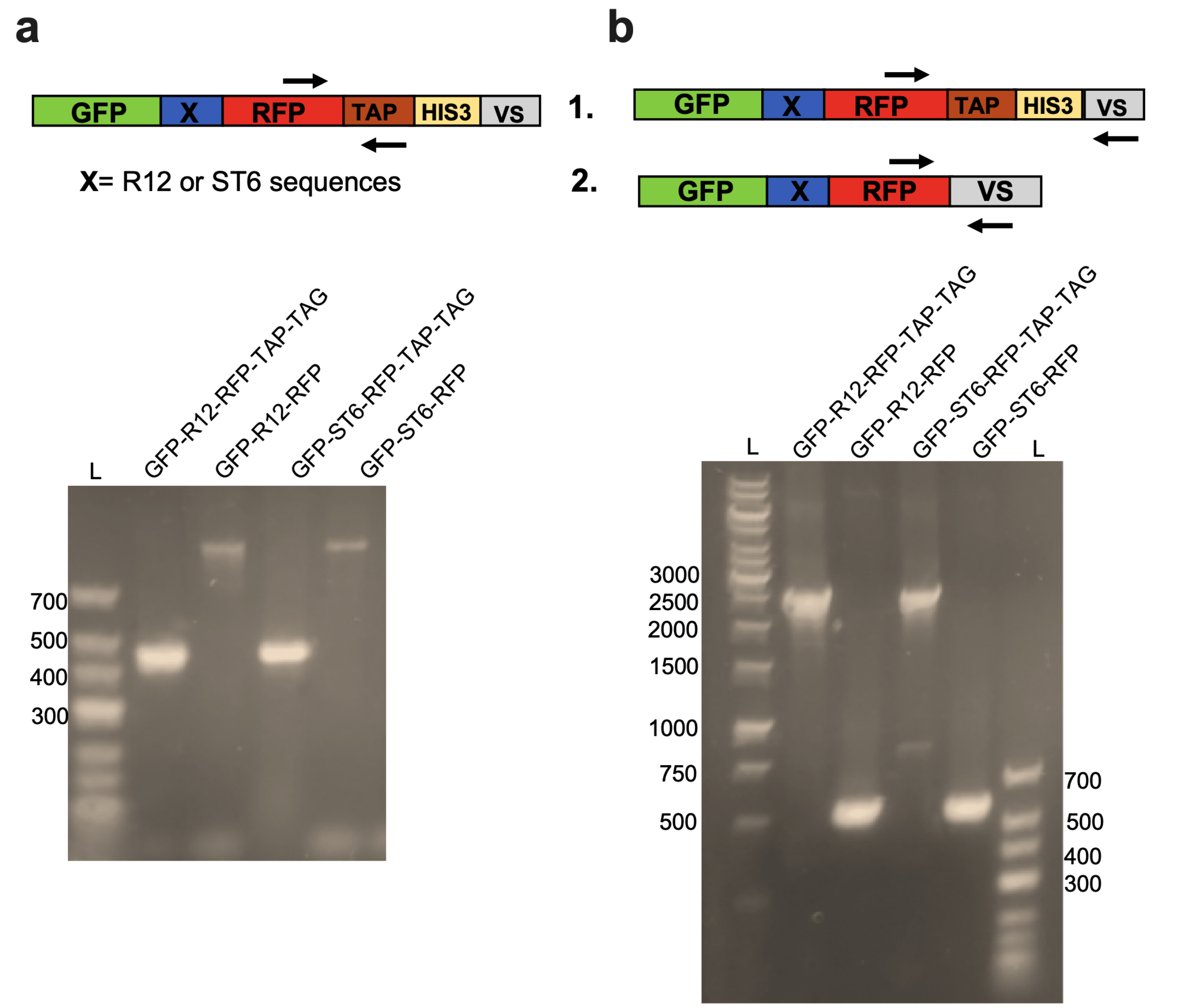

Figure S12. Confirmation of TAP-TAG insertion in GFP-R12-RFP and GFP-ST6-RFP constructs by PCR. The gels showed in the panels a and b confirm the TAP-TAG insertion in both reporters, GFP-R12-RFP and GFP-ST6-RFP. The two PCR sets shown the presence of the TAP-TAG in the 3' extremity of RFP as shown in the reporter scheme above the gels. (a) Visualization of an expected band between 400 and 500 bp in the constructs with the TAP-TAG insertion. (b) Visualization of a band close to 2500 bp corresponding to the insertion of the entire TAP-TAG sequence plus the His3Mx6 marker in the constructs (scheme 1). In the empty vector it was observed a band between 500 and 700 bp confirming the absence of the TAP-TAG sequence (scheme 2). VS= vector sequence, L= ladder, the numbers represent base pairs (bp).

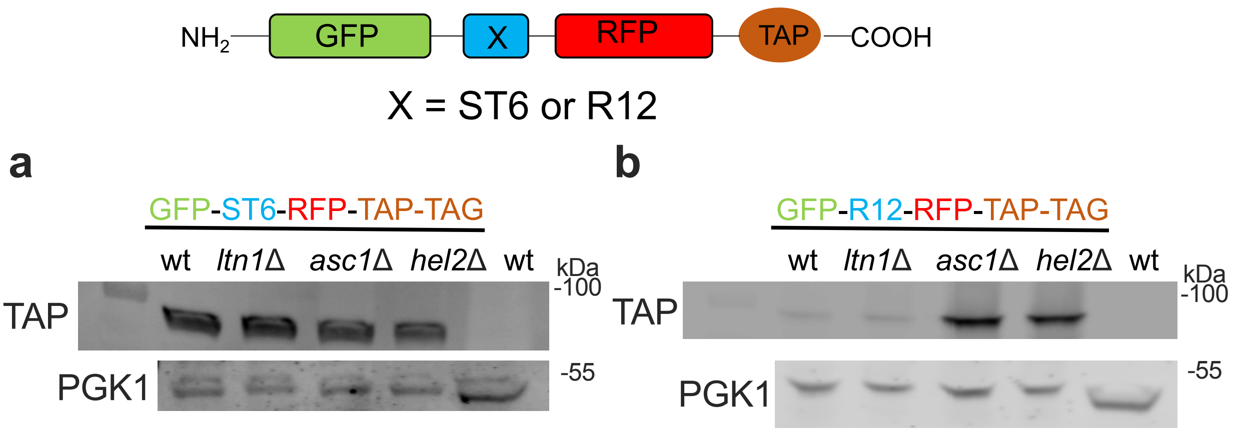

Figure S13. Whole-cell immunoblots of the indicated yeast strains expressing the non-stalling reporter (GFP-ST6-RFP-TAP-TAG) (a) and the stalling reporter (GFP-R12-RFP-TAP-TAG) (b). As a control for the antibodies labelling, we used a wt strain without reporters (last lane of each gel). The labelling with the anti-TAP antibody indicates the full-length translation product (GFP-RFP-TAP-TAG) that has approximately 75 kDa. The first lane of each TAP gel presents a marker with 100 kDa. ST6 means 6 serines + 6 threonines (non-stalling) while R12 means 12 arginines (stalling).

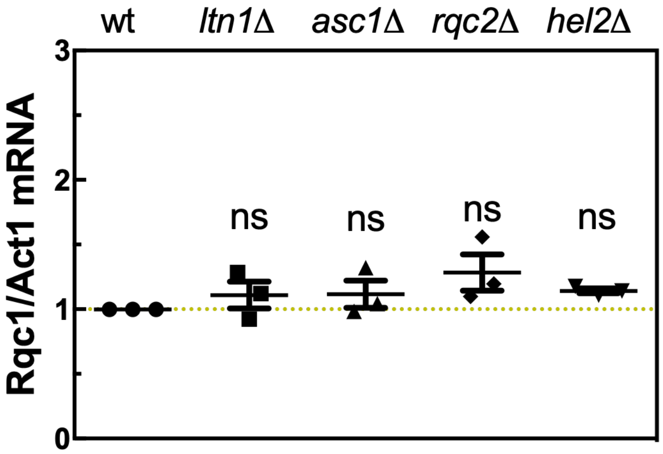

Figure S14. Non-tagged Rqc1 mRNA levels. qPCR was used to measure the non-tagged *RQC1* mRNA levels of the wt, *lnt1*Δ, *asc1*Δ, *rqc2*Δ, and *hel2*Δ strains. The *RQC1* levels were normalized in relation to the housekeeping gene actin (*ACT1*). The analysis of differential expression was made by relative quantification using $2^{-\Delta\Delta Ct}$ method. One-way ANOVA with Bonferroni's multiple comparison test was used for statistical analyses.

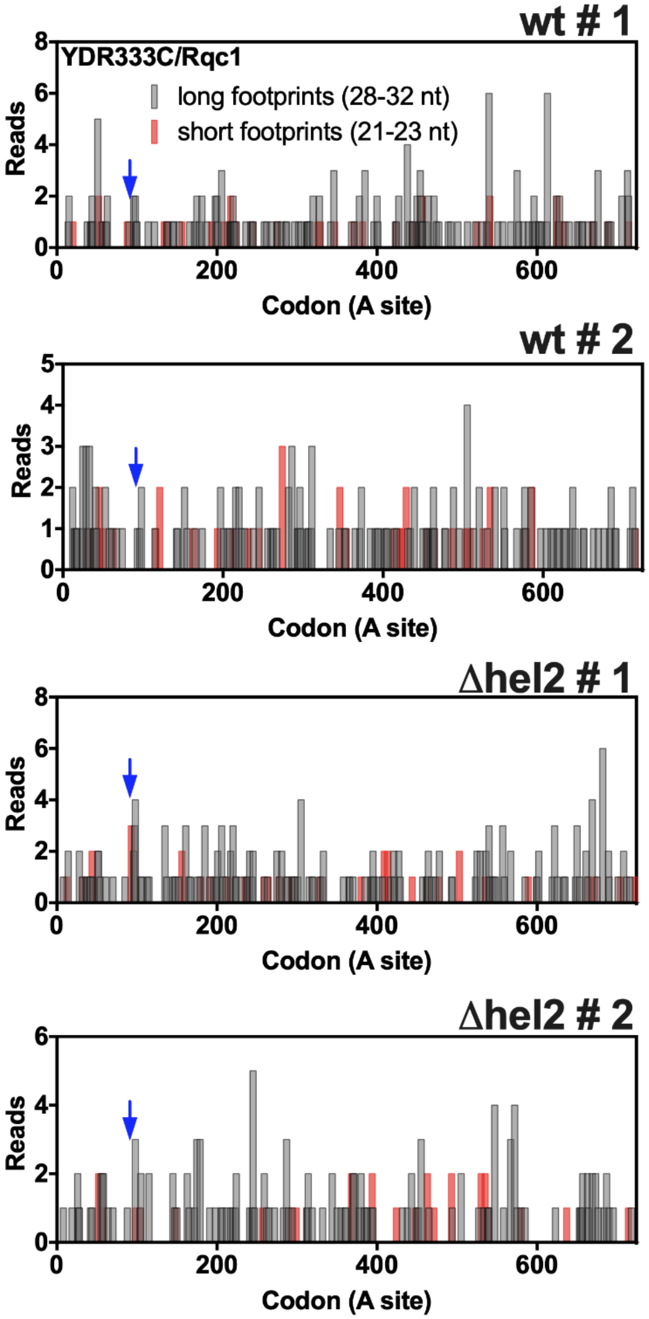

Figure S15. Ribosome profiling data of *RQC1*. 28-32 and 21-23 nucleotide footprint analysis of *RQC1*. Blue arrow indicates the start of the polybasic sequences. The reads were plotted at an approximate position of the ribosomal A site. The two upper panels represent ribosome profiling obtained with a wt strain while the two in the bottom were obtained with a *hel2*Δ strain.

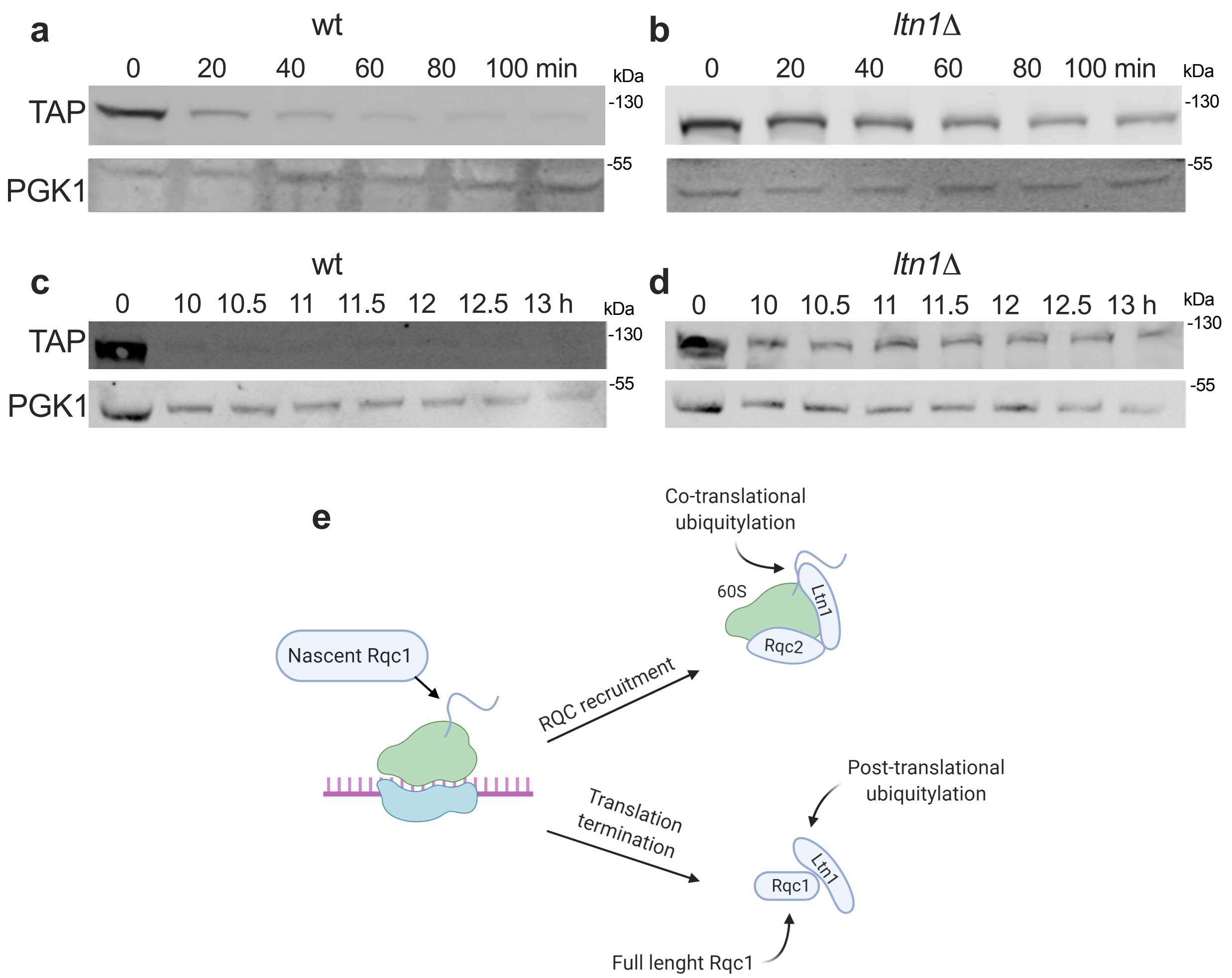

Figure S16. Rqc1 is post-translationally degraded by Ltn1. (a) Rqc1 protein expression diminishes over time in wt cells treated with 50 μg/ml of CHX (translation blocked). (b) In *ltn1*Δ cells treated with CHX Rqc1 half-life is partially stabilized. (c) and (d) show Rcq1 expression in wt and *ltn1*Δ cells, respectively, up to 13 hours CHX treatment. (e) Schematic model of Rqc1 degradation. The current model of Rqc1 degradation, proposed by Brandman *et al.*, 2012, says that Rqc1 is co-translationally degraded by the RQC (upper pathway). We proposed a different model, were Rqc1 is degraded post-translationally by Ltn1 (lower pathway); this model is supported by the observations that deletion of Ltn1 leads to the stabilization of full-length Rqc1 (Figure 5a) and that it is able to regulate Rqc1 expression even when there is no active translation (Figure S16 a-d).
